# Improving Methods for Analyzing Antimalarial Drug Efficacy Trials: Molecular Correction Based on Length-Polymorphic Markers *msp-1*, *msp-2*, and *glurp*

**DOI:** 10.1128/AAC.00590-19

**Published:** 2019-08-23

**Authors:** S. Jones, K. Kay, E. M. Hodel, S. Chy, A. Mbituyumuremyi, A. Uwimana, D. Menard, I. Felger, I. Hastings

**Affiliations:** a Department of Tropical Disease Biology, Liverpool School of Tropical Medicine, Liverpool, United Kingdom; b Metrum Research Group, Tariffville, Connecticut, USA; c Molecular & Clinical Pharmacology, University of Liverpool, Liverpool, United Kingdom; d Institut Pasteur in Cambodia, Phnom Penh, Cambodia; e Rwanda Biomedical Center, Gasabo, Kigali, Rwanda; f Malaria Genetics and Resistance Group, Biology of Host-Parasite Interactions Unit, Department of Parasites and Insect Vectors, Institut Pasteur, Paris, France; g Department of Medical Parasitology and Infection Biology, Molecular Diagnostics Unit, Swiss Tropical and Public Health Institute, Basel, Switzerland; h Centre for Drugs and Diagnostic Research, Liverpool School of Tropical Medicine, Liverpool, United Kingdom

**Keywords:** ACT, artemisinin, drug efficacy, drug failure, lumefantrine, malaria, mefloquine, modeling, molecular correction, piperaquine

## Abstract

Drug efficacy trials monitor the continued efficacy of front-line drugs against falciparum malaria. Overestimating efficacy results in a country retaining a failing drug as first-line treatment with associated increases in morbidity and mortality, while underestimating drug effectiveness leads to removal of an effective treatment with substantial practical and economic implications. Trials are challenging: they require long durations of follow-up to detect drug failures, and patients are frequently reinfected during that period. Molecular correction based on parasite genotypes distinguishes reinfections from drug failures to ensure the accuracy of failure rate estimates. Several molecular correction “algorithms” have been proposed, but which is most accurate and/or robust remains unknown. We used pharmacological modeling to simulate parasite dynamics and genetic signals that occur in patients enrolled in malaria drug clinical trials. We compared estimates of treatment failure obtained from a selection of proposed molecular correction algorithms against the known “true” failure rate in the model. Our findings are as follows. (i) Molecular correction is essential to avoid substantial overestimates of drug failure rates. (ii) The current WHO-recommended algorithm consistently underestimates the true failure rate. (iii) Newly proposed algorithms produce more accurate failure rate estimates; the most accurate algorithm depends on the choice of drug, trial follow-up length, and transmission intensity. (iv) Long durations of patient follow-up may be counterproductive; large numbers of new infections accumulate and may be misclassified, overestimating drug failure rate. (v) Our model was highly consistent with existing *in vivo* data. The current WHO-recommended method for molecular correction and analysis of clinical trials should be reevaluated and updated.

## Abstract (precis)

Drug efficacy trials monitor the continued efficacy of front-line drugs against falciparum malaria. Overestimating efficacy results in a country retaining a failing drug as first-line treatment with associated increases in morbidity and mortality, while underestimating drug effectiveness leads to removal of an effective treatment with substantial practical and economic implications.

## INTRODUCTION

**FIG 1:**
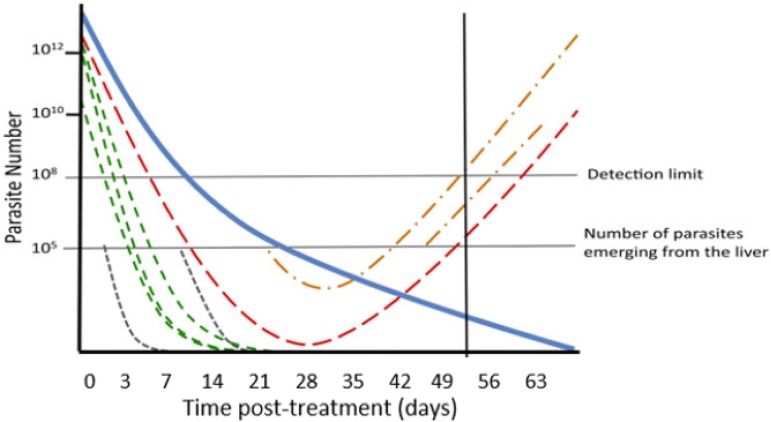
Malaria parasite dynamics following treatment of a hypothetical patient and the need for molecular correction (adapted from Jaki et al. [26]). Note that parasites only become detectable in the patient’s blood by light microscopy once their numbers exceed a detection limit at 10^8^ parasites. The blue solid line shows the declining concentration of drug posttreatment as it is eliminated by the patient’s metabolism. This patient had four malaria clones detectable at the time of treatment. The green lines represent initial clones that are cleared by the drug, and the red line represents an initial clone that recrudesces. Reinfections periodically emerge from the liver during follow-up in cohorts of ∼10^5^ parasites per clone. The gray lines are reinfections that are cleared by the drug. The orange lines are reinfections that are not cleared and survive to reach patency (i.e., increase in number to ≥10^8^, at which point they are detectable by microscopy). The solid black line is the point during follow-up at which the patient first has a patent recurrent infection (i.e., has a parasitemia sufficiently high that it is detectable by microscopy).

Malaria is endemic in over 100 countries and causes an estimated 400,000 deaths per annum (1); most deaths are caused by Plasmodium falciparum, and this study focuses on drug treatment of that species. Prompt treatment of malaria infections is an essential and effective public health tool, but drug resistance poses a constant threat to effective treatment of falciparum malaria. The World Health Organization (WHO) currently recommends that countries of endemicity test their first- and second-line antimalarial drugs every 2 years at sentinel sites to confirm their continued efficacy (2) and more frequently if resistance is suspected. The first-line treatments in most countries where malaria is endemic are artemisinin-based combination therapies (ACTs), consisting of an artemisinin component (artesunate [AS], artemether [AR], or dihydroartemisinin [DHA]), which rapidly clears parasites, and a “partner” drug that ensures eventual parasite clearance and therapeutic cure (3, 4). The clinical consequence is that malaria infections fall rapidly to undetectable levels immediately after ACT treatment initiation. The partner drugs (mefloquine [MQ], lumefantrine [LF], piperaquine [PPQ], amodiaquine, sulfadoxine-pyrimethamine, and pyronaridine) all have substantial half-lives. Infections surviving treatment are termed “recrudescences,” and many parasites may only recover to densities sufficiently high to become detectable once partner drug concentrations have decayed to ineffective concentrations—potentially weeks after treatment. Antimalarial drug efficacy studies therefore monitor patients for extended periods of time posttreatment to ensure recrudescences are detected. Duration of follow-up depends on the half-life of the drug being assessed (2, 5): usually between 4 and 6 weeks (28 to 42 days) (3), but sometimes extended to 9 weeks for research purposes. The critical operational problem is that new falciparum clones may be inoculated into patients by mosquitoes during these follow-up periods, and these infections (termed “reinfections”) must be distinguished from recrudescence to allow accurate estimates of drug efficacy (Fig. 1). This is not a trivial problem: annual entomological inoculation rates (aEIR) of malaria, a measure of malaria exposure in a population, are typically >10 and >100 per patient in areas of moderate to high transmission, respectively. Moderate- to high-transmission sites are preferred for clinical trials as morbidity from malaria is high, so trials cover the most-at-risk patient populations, and from a practical viewpoint, patient recruitment is straightforward.

The consensus method for distinguishing recrudescence from reinfections is molecular correction or, equivalently, PCR correction. A genetic profile of the malaria infection of each patient is taken just before treatment, with a second profile taken if the patient develops a detectable malaria infection during follow-up (known as “recurrent” parasitemia). If the profiles “match,” then the patient is considered to have a recrudescent infection, but if they do not match, the patient is considered to have a reinfection. This “matching” is simple in principle, but in practice has substantial limitations. The main problem is that individual malaria infections may consist of several genetically distinct clones. Current genotyping techniques struggle to detect minority clones that are present in relatively low numbers and/or which carry alleles that do not amplify well during the genotyping process. These limitations were recognized early in the development of molecular correction methodology (6–8) and led the WHO and Medicines for Malaria Venture (MMV) to cosponsor a meeting in 2007 to identify a consensus methodology for molecular correction; their findings were published in 2008 (3). Concerns surrounding the limitations of molecular correction have persisted (9, 10): previous studies have noted that different algorithms give different results when applied to clinical data (e.g., see Table 2 of reference 10), and a recent publication quantifying the limitations inherent in PCR detection has led to renewed calls for this methodology to be reexamined (11). There now exist several proposed sets of rules (referred to here as “algorithms”), for interpreting genetic profiles to classify patients (Table 1). The true failure rate is unknown *in vivo*, so it has been impossible to identify which algorithm is most accurate; consequently, the molecular correction field is currently in a state of limbo, with several alternative methods giving different results, but with no way of knowing which method is most accurate. Furthermore, some of these algorithms are newly proposed and have not been used to return failure rate estimates *in vivo*. There is a clear need for greater precision and improved harmonization in molecular correction techniques. Pharmacological simulation methods can be used to recreate data from clinical trials: since the true failure rate is known *in silico*, it is possible to quantify which algorithm provides the most accurate and/or robust method of analysis. The impact of drug efficacy trials is potentially enormous. Overestimates of drug efficacy may result in a country retaining a failing drug as first-line treatment with associated increases in morbidity and mortality, while underestimating drug effectiveness may lead to removal of an effective first-line treatment with substantial practical and economic implications.

**TABLE 1:** Molecular correction algorithms proposed to decide whether a patient presenting again with a recurrent malaria infection during follow-up is a recrudescence or a reinfection based on the WHO-recommended genetic markers of *msp-1*, *msp-2*, and *glurp*^*a*^

Algorithm	Reference	Definition	Consequences (identified in the model)
No correction		All recurrent infections are classified as recrudescence.	Algorithm grossly overestimates failure rate at higher FOI.^*b*^
WHO/MMV	2	Initial and recurrent samples must have shared alleles at all 3 markers to be classified as recrudescence.	Stringent conditions for recurrences to be classified as recrudescence mean that around 50% of true recrudescences are misclassified as reinfections, resulting in greatly underestimated failure rates. Most reinfections are correctly classified, so FOI has little impact on estimated failure rate.
No *glurp*	11	Results are as for the WHO/MMV algorithm but based on 2 loci (i.e., *msp-1* and *msp-2*). (*glurp* is omitted as it is prone to genotyping errors.)	Results are largely identical to the WHO/MMV method.
≥2/3 markers	11	Results are as for the WHO/MMV algorithm, but initial and recurrent samples must share alleles at least at 2 out of 3 markers to be classified as recrudescence.	Results are generally intermediate between no-*glurp* and allelic family switch algorithms.
Allelic family switch	11	Comparison was initially based on *msp-1* and *msp-2*. Identical alleles observed at both markers indicate recrudescence. Absence of shared alleles at both markers indicates reinfection. If 1 marker shares alleles and 1 does not (i.e., the sample is “discordant”), a complete allelic family shift in the nonsharing marker is required to classify a recurrence as a reinfection.	A tendency to misclassify reinfections as recrudescences leads to a dependency on FOI and results in large overestimates of failure rates at higher FOI, although the algorithm produces accurate failure rate estimates at low FOI.

a We also summarize the consequences of applying these algorithms for the analysis of clinical trials as quantified by our methodology: the failure rate estimates obtained from each algorithm are shown in Fig. 2 and Fig. 4.

b FOI, force of infection, our measure of transmission intensity. FOI is the mean number of malaria infections that emerge in an individual and would become patent in the absence of drug killing over the course of a year.

## RESULTS

We identified several types of misclassification of recurrent infections in our experiments:

1. Recrudescent infections could be misclassified as reinfection if the recrudescent allele(s) were not detected during the genotyping of the initial infection (i.e., for example, they were “minority alleles” [see Materials and Methods]).
2. A recrudescent infection could be misclassified as a reinfection if the recrudescent allele(s) were not detected during the genotype of the recurrent infection (i.e., for example, they were “minority alleles” [see Materials and Methods]).
3. A reinfection could be misclassified as recrudescent if it shares (by chance) alleles with clones present at time of treatment. The exact number (or type) of alleles that must be shared depended on the molecular correction algorithm chosen (i.e., the “no-*glurp*” algorithm was not affected by sharing an allele at *glurp*, and the “allelic family switch” algorithm was sensitive to sharing an *msp-1* or *msp-2* family by chance, whereas the other algorithms were not).

While not misclassification of recurrence, another source of bias affected the accuracy of failure rate estimates with respect to the true failure rate: a patient who failed to clear the initial infection may have had that infection persisting at a low level, below the limit of detection of detection of light microscopy (assumed, as described below, to be 10^8^ total parasites in all clones) and have no reinfection, such that parasites were never detected during follow-up (and thus, no recurrent sample was genotyped); this obviously depends on the duration of follow-up.

### Impact of algorithm choice on failure rate estimates.

Figure 2 shows the failure rates obtained from simulated DHA-PPQ clinical trials using four molecular correction algorithms and the noncorrected algorithm (Table 1), with a follow-up length of 42 days. Both the true failure rate and the estimated failure rate are presented (calculated using survival analysis) as a function of force of infection (FOI).

**FIG 2:**
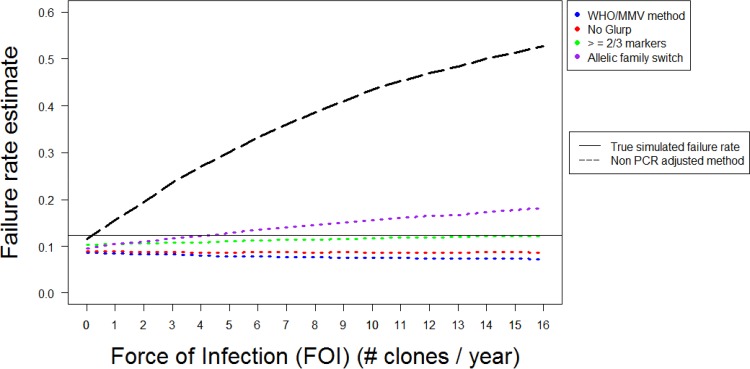
Analysis of simulated trial data for DHA-PPQ with a follow-up period of 42 days. Estimated failure rates are shown for the different algorithms of molecular correction (Table 1) as a function of force of infection (FOI) and are calculated using survival analysis. The multiplicity of infection (MOI) is drawn from data from Tanzania—a relatively high-transmission area.

The noncorrected algorithm always produced a higher failure rate estimate than any of the four molecular correction algorithms (Fig. 2). Failure rate estimates using no correction rose rapidly as FOI increased, and at moderate and high levels of transmission, estimated failure rates were substantially greater than the true failure rate. At high transmission intensities (FOI of 16), estimated failure rates produced by this algorithm were above 50%—a clear overestimate of the true failure rate (12%). This pattern occurred because all the additional reinfections that occurred as the FOI increased were misclassified as recrudescence. Conversely, in the absence of any reinfections (when FOI = 0), the noncorrected algorithm produced an accurate failure rate estimate by correctly classifying all recurrences as recrudescence (leaving only a slight underestimate due to patients who had recrudescent parasites at levels of <10^8^, such that no recurrence occurred during follow-up).

**FIG 3:**
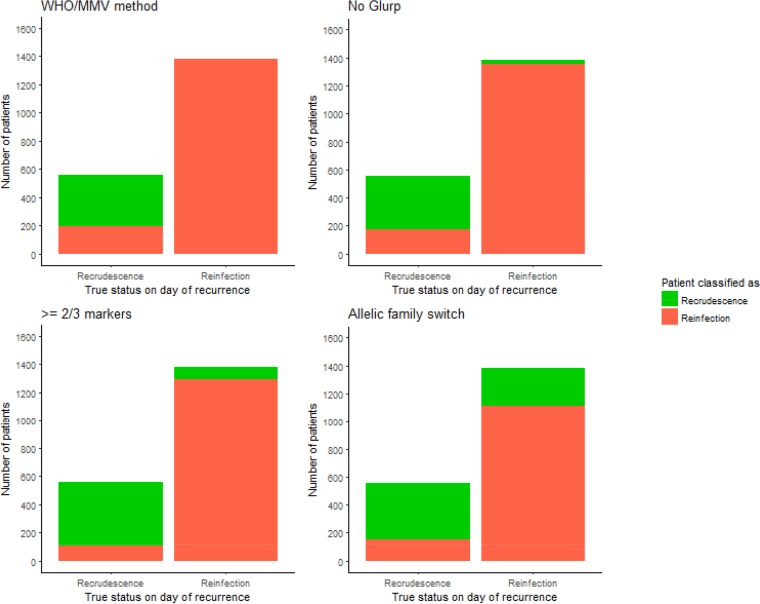
Figure showing the ability of the various molecular correction algorithms to correctly classify patients with recurrent malaria. The data are for DHA-PPQ with a 42-day follow-up obtained with an FOI of 8 (i.e., used to obtain the results shown at FOI = 8 in Fig. 2). The multiplicity of infection (MOI) is drawn from data from Tanzania—a relatively high-transmission area. The *x* axis shows the true status of patients on the day of recurrence (i.e., reinfection or recrudescence), and the color coding shows how these patients were classified by each algorithm. The WHO/MMV-recommended algorithm correctly classifies nearly all reinfections, but misclassifies around one-third of recrudescences. The no-*glurp* algorithm is similar to the WHO/MMV one; it misclassifies only a small number of reinfections, but misclassifies around a third of recrudescences. The ≥2/3 markers algorithm had fewer misclassifications and was also more balanced (i.e., misclassified similar proportions of both reinfections and recrudescences). Finally, the allelic family switch algorithm correctly classifies a large proportion of recrudescences but misclassifies around half of reinfections.

The ability of the four molecular correction algorithms to accurately estimate drug failure rates depended on their ability to correctly classify recrudescences and reinfections. This ability is shown (for an FOI of 8, i.e., a moderate-transmission area) in Fig. 3. Each algorithm misclassified some proportion of recrudescences and reinfections. The number of recrudescences misclassified as reinfections was consistent as the FOI changed, but the number of reinfections misclassified as recrudescence increased as the FOI increased—the results are shown in the supplemental material. (Note that while results for all parameterizations of AR-LF, AS-MQ and DHA-PPQ are not shown, the proportion of misclassification was extremely robust between drugs.) General trends were extremely clear:

• The WHO/MMV-recommended algorithm consistently underestimated failure rates at all transmission intensities as shown in Fig. 2. The algorithm frequently failed to detect drug failures (i.e., it misclassified around 40% of recrudescent infections as reinfections) (Fig. 3). These misclassifications occurred because of failure to detect recrudescent alleles in either the initial or recurrent blood sample: this algorithm was so stringent (requiring matching alleles at all three markers), that even missing a single allele could result in misclassification. As the FOI increased, the estimated failure rate did not change to any meaningful extent because the algorithm correctly classified nearly all reinfections (Fig. 3).
• The no-*glurp* algorithm produced slightly higher estimated failure rates than the WHO/MMV algorithm across all FOI settings (Fig. 2). This occurred because recrudescences were slightly less likely to be misclassified as reinfections, while reinfections were slightly more likely to be misclassified as recrudescences than under the WHO/MMV algorithm (Fig. 3). At low FOI, this difference was small; the high allelic diversity of *msp-1* and *msp-2* meant misclassification of reinfections as recrudescences was rare. The difference between the no-*glurp* algorithm and the WHO/MMV algorithm increased as FOI increased, but like the WHO/MMV algorithm, the no-*glurp* algorithm always underestimated the true failure rate.
• The “≥2/3 markers” algorithm produced higher estimated failure rates than the no-*glurp* algorithm across all FOI levels. This occurred because this algorithm reduced the chance of a recrudescence being misclassified as a reinfection (due to failure to detect recrudescent alleles) and increased the chance of a reinfection being misclassified as a recrudescence (Fig. 3). Both effects occurred because only needing matching alleles at 2/3 markers gave the algorithm some tolerance to undetectable alleles.
• The “allelic family switch” algorithm produced higher estimated failure rates than the ≥2/3 markers algorithms at all but the lowest FOI settings (0 to 2) (Fig. 2). A complete family switch in *msp-1* or *msp*-2 in a discordant sample (Table 1) would be sufficient to classify a recrudescence. This led to a similar number of recrudescences being correctly classified by the ≥2/3 markers algorithm, but this algorithm misclassified the largest number of reinfections as recrudescences out of all the molecular correction algorithms—the family switch could still occur (by chance). The difference in numbers misclassified between the no-*glurp* algorithm and the allelic family switch algorithm is the result of this misclassification by chance.

### Impact of follow-up length on failure rate estimates.

**FIG 4:**
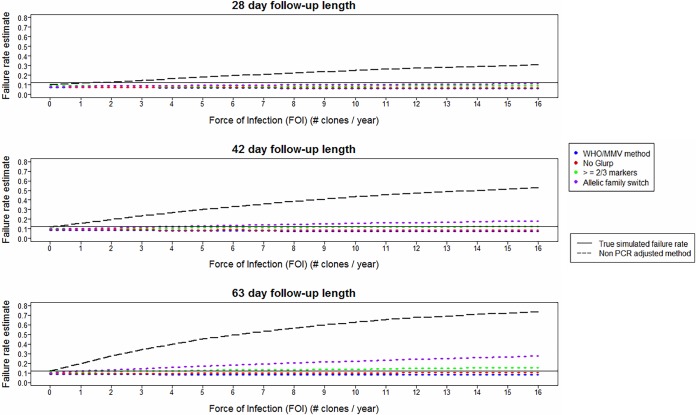
Analysis of simulated trial data for DHA-PPQ showing the impact of changing follow-up period with follow-up lengths of 28 days, 42 days (as in Fig. 2), and 63 days. Estimated failure rates are shown for the different molecular correction algorithms (Table 1) as a function of FOI and calculated using survival analysis. The multiplicity of infection (MOI) is drawn from data from Tanzania—a relatively high-transmission area.

Alternate durations of follow-up length were simulated for DHA-PPQ, and their impacts on estimated failure rates are shown in Fig. 4 for 28, 42, and 63 days of follow-up. Longer durations of follow-up led to larger estimated failure rates for all algorithms. This occurred because longer follow-up (i) allowed more time for recrudescences to become detectable and (ii) allowed more reinfections to emerge, some of which were misclassified as recrudescences (Fig. 3).

**FIG 5:**
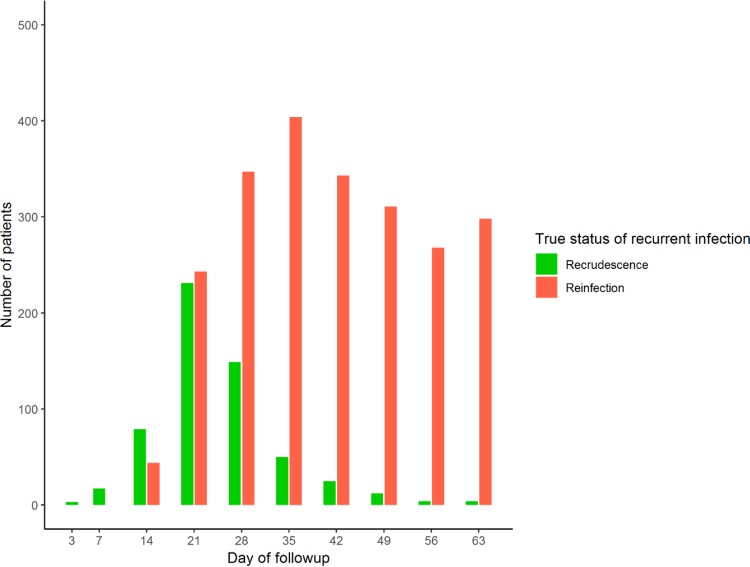
The true status of recurrent infections on each day of follow-up for a simulated trial of DHA-PPQ with a true failure rate of 12% and an FOI of 8. The multiplicity of infection (MOI) is drawn from data from Tanzania—a relatively high-transmission area. The total height of the bars indicates the number of recurrent infections detected on that day of follow-up, and the color coding shows the number of those recurrent infections that were truly recrudescent or reinfections.

Underestimation of the true failure rate occurred with all algorithms when a 28-day follow-up period was chosen. With a 42-day follow-up period, the allelic family switch algorithm produced the most accurate failure rate estimate, with an FOI of <7, and the ≥2/3 markers algorithm produced the most accurate failure rate estimate, with an FOI of ≥7. As length of follow-up increased to 63 days, the ≥2/3 markers algorithm tended to slightly overestimate the failure rate. This effect was more apparent as the FOI increased. These patterns emerged because only a small number of initial clones recrudesced after 42 days. Figure 5 shows the proportion of recurrent infections on each day of the follow-up period that were truly recrudescent or reinfections. On days 49, 56, and 63, the number of recurrent infections that were truly recrudescent was small. Almost all recurrent infections on these days were reinfections, and consequently, inclusion of these three extra days of follow-up inflated the estimated failure rate due to misclassification of these reinfections as recrudescences (as alleles were shared by chance between these reinfections and the initial blood sample). However, the increased failure rate of a 42-day follow-up compared to a 28-day follow-up (due to both detection of true recrudescences and misclassification of extra reinfections) meant that a 42-day follow-up period analyzed with either the ≥2/3 markers or allelic family switch algorithm produced more accurate failure rate estimates than the WHO/MMV algorithm.

### Results for other drugs/parameterizations/model settings.

Additional models for other ACTs are described in the supplemental material. In brief, these drugs differed from DHA-PPQ mainly in their persistence of active drug concentrations posttreatment (and hence in their prophylaxis against reinfections). Results for failing AR-LF and AS-MQ were highly consistent with those described above for DHA-PPQ, showing the same qualitative patterns (i.e., that failure rate estimates increase as FOI increases and as the follow-up period increases, that the WHO/MMV algorithm underestimates, that no correction leads to large overestimates, and that the ≥2/3 markers algorithm was generally accurate across a range of FOI values).

Different prophylactic profiles meant that the most effective duration of follow-up for AR-LF and AS-MQ (as would be expected) differed from that for DHA-PPQ; using the ≥2/3 markers molecular correction algorithm, a 28-day follow-up for AR-LF appeared to be most accurate at moderate to high FOI. A 49-day follow-up for AS-MQ appeared to be the most accurate with the ≥2/3 markers algorithm, but increased accuracy over the WHO/MMV algorithm was also seen with shorter follow-up periods.

Models of nonfailing (i.e., clinically effective) pharmacokinetic/pharmacodynamic (PK/PD) calibration of AR-LF and AS-MQ showed that the ≥2/3 markers algorithm slightly underestimated the true failure rate, but this difference was small, and there is no evidence that this algorithm would incorrectly identify effective drugs as failing.

Alternative parasite dynamics for DHA-PPQ were generated using a three-compartment model with PK parameters described in reference 12 to reflect to uncertainty around how PPQ should be modeled. (We previously identified and analyzed 6 published and distinct PK calibrations for piperaquine [13]; note that PD parameters remained as for the two-compartment assumption, as described in the supplemental material.) Parasite dynamics obtained using this three-compartment calibration resulted in a more prophylactic drug (i.e., fewer reinfections became patent) with a lower true failure rate (10% with unchanged PD parameters). The relative failure rate estimates of the algorithms and the no-correction approach were the same—i.e., that the WHO/MMV algorithm produces the lowest failure rate estimate, followed by the no-*glurp*, ≥2/3 markers, and allelic family switch algorithms. Failure rate estimates are lower across all algorithms than with the shorter-prophylaxis two-compartment model, and a 63-day follow-up appears to be the most suitable under this calibration: the ≥2/3 markers algorithm produced an accurate failure rate estimate at all but the lowest FOI levels with this follow-up length. Crucially, the key message is the same: the WHO/MMV algorithm underestimates the true failure rate, and other algorithms can produce more accurate failure rate estimates. Perhaps the most interesting difference between the two DHA-PPQ PK/PD calibrations is that they suggested, given use of the same molecular correction algorithm, different optimal lengths of follow-up.

Failure rate estimates were calculated using the per protocol method rather than survival analysis. The per protocol method led to increased failure rate estimates with all algorithms, all ACTs, and all follow-up periods. These results are discussed in the supplemental material.

Finally, the simulation was validated by varying the multiplicity of infection (MOI) at time of treatment, the relative detectability of alleles based on length, and the minority allele detection threshold. The results of these analyses are provided in the supplemental material and showed mostly the same qualitative patterns as the results presented above; the one key departure was that assumption of a minority allele threshold of 5% (reduced from the assumption of 25% presented above) led to slightly increased failure rates and the no-*glurp* algorithm being the most accurate at a moderate to high FOI.

### Reanalysis of clinical data.

Clinical data from Rwanda (a relatively high-transmission area) were reanalyzed using the proposed molecular correction algorithms (Table 2) and were highly consistent with our models: i.e., the WHO/MMV algorithm produced the lowest estimated failure rate, followed by the no-*glurp* algorithm, the ≥2/3 markers algorithm, then the allelic family switch algorithm. The pattern was quantitatively consistent: the WHO/MMV algorithm estimated failure rates to be around half that obtained by the ≥2/3 markers algorithm. Results are similarly consistent with reanalysis of a trial from low-transmission settings in Cambodia (Table 2). The impact of algorithm choice was not so large in Cambodia because the FOI was low: 62 of the recurrences had matching alleles at all 3 loci and so were presumably drug failures and would have been classified as such by all four algorithms. There were only 3 potential reinfections (all following DHA-PPQ treatment): one patient had no shared alleles at any locus and so was classified as a reinfection under all four algorithms, but the other two patients shared alleles at both *msp-1* and *msp-2* and were only classified as reinfections under the WHO/MMV algorithm because no common alleles were noted at *glurp*. In contrast, the other algorithms all classified both patients as being drug failures. In summary, as in the high-transmission data, the WHO/MMV algorithm had a higher tendency to classify recurrences as reinfections compared to the other algorithms. Note also that, consistent with Fig. 4, the choice of algorithm makes little operational difference at low FOI: using the WHO/MMV algorithm identified 62 drug failures and three reinfections, while the other algorithms give 64 drug failures and 1 reinfection, a negligible increase in number of drug failures.

**TABLE 2:** Molecular correction with multiple algorithms from reanalysis of clinical trial data from Rwanda (a high-transmission study site) and Cambodia (a low-transmission site)^*a*^

Country	Drugs tested^*b*^	Classification of recurrent infection	No. of infections classified by algorithm			
			WHO/MMV	No *glurp*	≥2/3 markers	Allelic family switch
Rwanda	AR-LF	Recrudescence	17	27	36	59
		Reinfections	93	83	73	51
	DHA-PPQ	Recrudescence	3	6	8	18
		Reinfections	40	37	35	25

Cambodia	AS-AQ	Recrudescence	5	5	5	5
		Reinfections	0	0	0	0
	DHA-PPQ	Recrudescence	45	47	47	47
		Reinfections	3	1	1	1
	AS-PYN	Recrudescence	12	12	12	12
		Reinfections	0	0	0	0

a Full details of study sites and methodology are provided in Materials and Methods.

b AS-AQ, artesunate plus amodiaquine; DHA-PPQ, dihydroartemisinin plus piperaquine; AR-LF, artemether plus lumefantrine; AS-PYN, artesunate plus pyronaridine.

Finally, we reviewed clinical trials that reported failure rates based on no correction and the WHO/MMV algorithm (Table 3). The magnitudes of differences in failure rate estimates were similar to those noted in the results from our model, where the noncorrected algorithm and the WHO/MMV algorithm produced the highest and lowest failure rate estimates, respectively.

**TABLE 3:** The need for molecular correction shown by a comparison of estimated drug failure rates obtained without correction versus with molecular correction performed according to the current WHO/MMV-recommended algorithm^*a*^

Drugs tested^*b*^	Uncorrected vs corrected failure rates	Country, yr	Reference
AR-LF	54% vs 10%	Burkina Faso, 2014	36
AS-AQ	42% vs 10%	Burkina Faso, 2014	36
AS-AQ	17% vs 6%	Congo, 2013	37
AR-LF	22% vs 0%	Tanzania, 2014	38
AR-LF	13% vs 0%	Benin, 2016	39
AR-LF	9% vs 2%	Mozambique, 2015	40
AR-LF	2% vs 1%	India, 2015	41
AR-LF	16% vs 1%	Congo, 2012	42
AS-AQ	22% vs 5%	Congo, 2012	42

a Failure rate was calculated as 1 − the 28-day adequate clinical and parasitological response reported in the study (from data collated and provided by Jörge Möhrle and Stephan Duparc).

b AR-LF, artemether plus lumefantrine; AS-AQ, artesunate plus amodiaquine.

## DISCUSSION

The key message presented here is that none of the proposed algorithms using *msp-1*, *msp-2*, and *glurp* correctly classified all recurrent infections (Fig. 3), nor is it likely that such an algorithm exists due to the limitations of the PCR correction process (11). The ability of each algorithm to accurately recover the true failure rate was dependent on the transmission intensity (quantified in these models by FOI) due to the different propensity of each algorithm to misclassify reinfections as recrudescence (which occurred when alleles are shared by chance or a clone that later recrudesces was not observed in the initial sample) (Fig. 3). The 2-fold underestimation of true failure rates that occurred at all FOI levels using the current WHO/MMV algorithm is a cause for considerable concern. This underestimate occurred because this algorithm was extremely stringent—it did not misclassify any reinfections as recrudescence (Fig. 3) but did misclassify some recrudescences as reinfections when a clone that later recrudesced was not detected in the initial sample (due to the issues inherent in the PCR methodology with detecting minority alleles and longer alleles). These issues are shared between algorithms; however, the no-*glurp*, ≥2/3 markers, and allelic family switch algorithms are all less stringent and misclassified some reinfections as recrudescences (Fig. 3), which increased failure rate estimates and accounted—to some extent—for the underestimation of failure rates.

Key to identifying a methodology that gives consistently accurate estimated failure rates is to minimize and balance errors that arise from molecular correction, which are in turn, influenced by factors including FOI, duration of follow-up, and sensitivity of the PCR protocols. Despite these concerns, these results show that operationally important increases in accuracy of estimated failure rates for antimalarial efficacy trials are achievable with alternate genotyping algorithms. It is undesirable to recommend different molecular correction algorithms for different ACTs and transmission intensity levels (as this would be likely to cause confusion): hence, the approach of investigating multiple ACTs and various transmission intensities through FOI to assess if a single algorithm may be identified that gives robust and accurate estimates. Based on the results presented here, it appeared that the ≥2/3 markers algorithm was the most robust in areas of moderate to high transmission and provided estimated failure rates close to (typically within 2 percentage units) the true failure rate (Fig. 2 and 4; supplemental material).

The other factor that can affect estimates of drug efficacy, given that molecular correction is imperfect, is the duration of follow-up. Recommended duration has gradually increased over the last 20 to 30 years, with the objective of capturing all (or at least the majority of) recrudescences. However, the objective of clinical trials is not to capture every recrudescence, but to obtain accurate and robust estimates of efficacy. Figure 5 shows that in areas of moderate to high FOI, the penalty for detecting the last few recrudescences by extending the follow-up period was the inclusion of a much larger number of reinfections. These reinfections inflate the estimated failure rate due to the propensity of molecular correction algorithms to misclassify some reinfections as recrudescences (Fig. 3). It is obviously preferable to have the shortest follow-up possible while retaining the accuracy of failure rate estimates: based on the results shown in Fig. 4 and analogous plots for failing AR-LF and AS-MQ (supplemental material), using the ≥2/3 markers algorithm provided accurate estimates with a follow-up of 28 days for AR-LF, 42 days for DHA-PPQ, and 49 days for AS-MQ, which are all roughly in line with current WHO recommendations (2, 3). Importantly, the accuracy of the estimates with this algorithm appeared to be relatively robust to changes in transmission intensity, quantified in these models by FOI. (The WHO/MMV and no-*glurp* algorithms were also robust to changes in FOI, but had an underestimate of failure rate associated with them.) Note that a different DHA-PPQ parameterization (i.e., one that is more prophylactic [supplemental material]) favored a longer follow-up period more in line with MQ, which also has longer prophylaxis posttreatment. The trends across all drugs modeled are clear: it is highly likely that use of the current WHO/MMV algorithm will generate substantial (near 2-fold) underestimates of failure rates and that switching to an alternative correction algorithm should be considered a matter of urgency.

Technical problems with molecular correction approaches exist (identified and explained in, for example, references 9 and 11), which gives rise to the temptation to simply ignore molecular correction and just use uncorrected data. The results presented here strongly suggest that appropriate use of molecular correction is essential. Trials conducted in areas of moderate to high transmission intensity, which are the areas where most malaria morbidity and mortality occur, analyzed without molecular correction will lead to severe overestimates of the true failure rate. This assertion is supported by clinical data (Table 3), which clearly show that large discrepancies may arise in the absence of molecular correction. Ignoring molecular correction (i.e., non-PCR-corrected algorithm in Fig. 2 and 4) only produced accurate estimates of failure rates when the FOI was very low (a fact generally acknowledged in the literature [2, 3]). However, caution must be taken even when using no correction in “low-transmission” areas. Malaria transmission is highly focal, and even if an area is, on average, very low transmission, it is plausible that most patients will be recruited from foci of high transmission, where FOI may well be sufficient to invalidate estimates based on no correction.

The evaluation of different classification algorithms relied on simulated data. This was not ideal, but there is no obvious alterative given that key parameters (including the vital one—true failure rate), cannot be directly observed *in vivo*. Confidence in this approach was ensured given the past success of pharmacological modeling to correctly reflect and predict clinical data (e.g., see references 4 and 14 to 18) and the consistency of the simulated results with *in vivo* Rwandan and Cambodian data sets. We acknowledge that our model may not reflect the *in vivo* parameters of these trials (though see the discussion for the parameter space we covered in the supplemental material); however, the purpose of reanalysis of these data was to investigate the change in failure rates from use of proposed algorithms on *in vivo* data—analysis of trial results with these algorithms had not previously taken place. This reanalysis is not dependent on our model parameter space (nor vice versa), and all algorithms require the same data (the *msp-1*, *msp-2*, and *glurp* alleles [and families for the former two]); consequently, this reanalysis showing similar trends to our modeled results is encouraging.

Focus has been on the current WHO-recommended marker loci *msp-1*, *msp-2*, and *glurp* and how they may be best used to distinguish recrudescences from reinfections; it would be straightforward to repeat these analyses for different types of molecular data, such as deep-sequenced amplicons, microsatellites, and single nucleotide polymorphism (SNP) barcodes, and this is discussed further in the supplemental material. Notably, reduction of the minority detection threshold to 5% increased the failure rate estimates and altered which algorithm produced the most accurate estimate. We are confident that the length-polymorphic markers do not have this level of sensitivity. We analyzed this assumption solely to test its effect on our results; however, this threshold emulates more closely the use of amplicon sequencing, where minority alleles are easier to detect, and we intend to test the accuracy of failure rate estimates with amplicon sequencing using a similar methodology in the future.

There is concern in the literature that reinfections may share alleles with the initial infection purely by chance and that subsequent misclassification of reinfections as recrudescence would lead to overestimation of failure rates (9). This could arise in areas of high transmission (7) as increased MOI leads to more alleles in the initial sample; these can later be shared with a reinfection purely by chance. This could also occur in low-transmission areas, where genetic diversity is lower and there is more chance of a match by chance. Importantly, we did not observe large-scale overestimation (e.g., the low impact of FOI on estimated failure rate using the ≥2/3 markers algorithm in Fig. 2 and supplemental material) with increased transmission intensity with either a high MOI (Fig. 4) or a low MOI (supplemental material), suggesting these fears are unlikely to have a large impact in practice.

In conclusion, both our modeling approach and reanalysis of clinical data suggest that more accurate and easily implemented algorithms are available to analyze clinical data and the field should consider implementing these methods. Which algorithm will perform best will depend on factors in the patient population/area—our results demonstrate this explicitly for transmission intensity (FOI) and follow-up length. The four algorithms investigated here are not mutually exclusive and are based on the same data. Our firm recommendation is that initial and recurrent samples should be genotyped at all three loci: when using the current WHO/MMV algorithm, there is no need to genotype after a mismatch has occurred at one locus, so genotyping is often incomplete. These complete data would allow results obtained from all four algorithms to be presented; this maintains consistency with previous analyses based on the WHO/MMV algorithm, while also providing results that are likely to provide a substantially more robust estimate of malaria drug clinical failure rates.

## MATERIALS AND METHODS

The World Health Organization (WHO) has published a standardized “consensus” list of terms (19) that we have used throughout this work, with a key exception: our key term “drug failure” is not equivalent to “treatment failure” because according to the WHO definitions, late treatment failure (LTF) includes patients who either failed drug treatment (i.e., recrudescence) or acquired a reinfection during follow up (2, 19). The unambiguous term “drug failure” will be used here to indicate that a patient’s initial infection was not cleared by drug treatment.

To create a model with which to investigate the accuracy of molecular correction methods, we used a two-stage process implemented in the statistical programming language R (version 3.5.1) (20):

1. Use pharmacological modeling to simulate the parasite dynamics posttreatment in a population of patients enrolled in a clinical trial and track subsequent intrahost P. falciparum dynamics in these patients posttreatment.
2. Allocate genetic signals to each simulated parasite clone and calculate the genetic signals detected from a patient’s blood sample at a given follow-up day (dependent on a variety of factors, as explored later), then analyze these signals using different algorithms (Table 1) to classify recurrent infections as drug failures or reinfections. This classification was used to generate drug failure rate estimates with comparison to true drug failure rates, thus, determining if improvements in the accuracy of these estimates were obtainable through adoption of novel algorithms.

Malaria parasite dynamics were generated using pharmacological models of malaria drug treatment that have been developed over the last decade (e.g., see references 14 to 17 and [Bibr B21][Bibr B22][Bibr B23]) and previously calibrated and validated for three front-line ACTs: dihydroarteminisin-piperaquine (DHA-PPQ), artemether-lumefantrine (AR-LF), and artesunate-mefloquine (AS-MQ). The key advantage of this approach was that the exact parasitemia of each malaria clone in each simulated patient at each time point posttreatment was known (Fig. 1), as was the true status (i.e., recrudescent or reinfection) of any recurrent infection that occurred in that patient. This allowed testing of how well different PCR correction algorithms classified recurrent infections as “drug failures” or “reinfections.” It also meant the true failure rate of the drug in the simulated trial was known, as this could be calculated directly from models of parasite dynamics: i.e., did all the initial clones clear by the final day of follow-up? This allowed each algorithm to be tested for accuracy against the true failure rate in the simulation.

### Generation of parasite dynamics posttreatment using PK/PD models.

Parasite dynamics were generated using existing pharmacokinetic/pharmacodynamic (PK/PD) models; these models have been calibrated and validated for a range of ACTs and successfully used to investigate a variety of key research questions (13–18, 21). The PK/PD parameters used to generate these dynamics for each ACT are described in full in the supplemental material. It is important to note that our results were not dependent on any choice of calibration. A full discussion of the variation that use of different PK and PD parameters would induce in our results is included in the supplemental material. Variation was included for all parameters, and we later show that our findings with regard to the relative performance of the molecular correction algorithms were consistent across three different ACTs, multiple PD parameterizations (i.e., changing the 50% inhibitory concentration [IC_50_] to simulate failing/nonfailing drugs), and for both a two-compartment model and three-compartment model of DHA-PPQ (explored extensively in the supplemental material). Thus, we are confident that PK/PD models of DHA-PPQ, AR-LF, and AS-MQ were appropriate means by which to generate parasite dynamics posttreatment for the purposes of this study. Alternative methods were available: i.e., arbitrarily constructing recurrent infections as containing a given proportion of recrudescent and/or reinfection and testing the algorithm’s ability to correctly classify them (as is routinely done to construct laboratory mixtures [e.g., see reference 11]) or setting distributions of time until recrudescence and/or reinfection and using these distributions to construct recurrent infections. However, the use of an explicit PK/PD model added an additional level of realism to these arbitrary approaches: it was simple, easily scalable, more realistic, and allowed for future tuning and testing if novel parameterizations emerge within the field for these and for other antimalarial drugs.

While it was obviously not feasible for us to simulate and present every possible parameterization to create parasite dynamics likely to occur in trials (though note our included variation covers a large range of possible values), calibrating the models to rerun a specific set of parameters for interested groups is a simple task upon provision of the parameters.

### Number of malaria clones per patient.

A malaria infection may consist of several genetically distinct parasite clones, and the number of clones in a patient at the time of treatment is termed the multiplicity of infection (MOI). Two MOI distributions were used in our models. A “high MOI” is representative of the MOI in an area of intense transmission—in this case, Tanzania, where MOI of 1 to 8 were assigned with probabilities of 0.036, 0.402, 0.110, 0.110, 0.183, 0.049, 0.061, and 0.049, respectively (25). A “low-MOI” distribution was based on data from Papua New Guinea, with probabilities of 0.460, 0.370, 0.150, and 0.020 for MOI of 1 to 4, respectively (26); these two distributions were used to check if the accuracies of different algorithms were consistent across different MOI. Each clone within the MOI (later called “initial clones”) had its starting parasitemia drawn from a log-uniform distribution spanning from 10^10^ to 10^11^ asexual parasites per person. Previous modeling approaches (26) used 10^12^ parasites as the upper limit of parasitemia because this level of parasitemia is likely to be lethal or at least be a parasite density sufficiently high that such patients would not be enrolled in a clinical trial; hence, 10^11^ was used as the upper limit for any single clone at the time of treatment.

Reinfections emerging from the liver are illustrated as the gray and orange dotted lines in Fig. 1. Reinfections were assumed to emerge from the liver with a parasitemia of 10^5^, and all drugs modeled were assumed to be inactive against the hepatic stages. The rate of emergence reflected the local intensity of malaria transmission and was quantified as the “force of infection” (FOI). At the start of the model, each patient was assigned the number of reinfections that would emerge during a year. This number was drawn from a Poisson distribution whose mean value was the FOI. Values for FOI from 0 to 16 were used to reflect low-, medium-, and high-transmission areas; as a general guide, we regarded FOI ≤ 2 as representing a low-transmission setting, 2 < FOI ≤ 8 as indicative of moderate transmission intensity, and FOI > 8 as high transmission; the yearly value was then converted to the number of reinfections occurring during the follow-up period. (See the supplemental material for a detailed discussion of FOI values.)

### Tracking parasite numbers (parasitemia) over time.

Multiple lengths of follow-up are permitted in the WHO guidelines (3) and used in practice (27). The length of the follow-up period affects drug failure rate estimates in two ways: First, a longer follow-up period will allow more time for recrudescent clones to become detectable (i.e., if a patient had parasites that would recrudesce and become detectable on day 60 and the follow-up period was 28 days, this recrudescence would not be observed). Second, a longer follow-up period leads to more reinfections emerging in each patient, some of which may be misclassified as recrudescence and inflate failure rate estimates. Accurate, robust analyses need to balance these two risks through appropriate choice of follow-up duration. WHO guidelines (2) stipulate that patients be checked for recurrent parasitemia by light microscopy on scheduled days of follow-up. A 28-day follow-up schedule requires patients be examined on days 3, 7, 14, 21, and 28. A 42-day follow-up period uses two additional days: i.e., days 35 and 42. A 63-day follow-up period (not recommended in routine surveillance) has scheduled visits as per the 42 days but with 3 extra days, i.e., days 49, 56, and 63. Novel lengths of follow-up were simulated simply by “ending” the trial on any given day of follow-up: i.e., to investigate a 35-day follow-up length, patients were checked on days 3, 7, 14, 21, 28, and 35.

The parasitemia of each clone in each patient was tracked and updated each day to reflect two factors. First was the extent of drug killing based on the PK/PD parameters (see calibration of PK/PD parameters in Table S1 in the supplemental material); second was the growth rate of each clone, which was assumed to be identical for every clone and set to 1.15/day as in previous modeling work (14, 28). The model assumed that if the total parasitemia (i.e., the sum of parasitemia of all clones) in a patient at any time reached 10^12^, then density-dependent effects, such as fever, set the growth rate of every clone to 0.

The model checked each day of scheduled follow-up to determine whether a patient had enough parasitemia that a recurrence would be detectable by light microscopy—parasitemia was considered detectable if the total number in a patient was ≥10^8^ on that day. We note that variance in the limit of detection by light microscopy exists with respect to the skill of the microscopist (29); we have chosen here to assume this limit reflective of an “expert” microscopist (corresponding to roughly 20 parasites/μl of blood).

### Allocation and analysis of genetic data.

Each clone, whether an initial clone present at treatment or a reinfection that emerged during the follow-period, was assigned a genetic profile based on three markers: *msp-1*, *msp-2*, and *glurp*, using previously established distributions for the frequency of alleles. *msp-1* and *msp-2* allelic frequency distributions and amplicon sizes were derived from 115 or 108 patients from Tanzania (30). *glurp* distributions were drawn from a collection of field samples described in reference 11. The length of each allele and its allelic family (for *msp-1* and *msp-2*) were also noted. The distributions we used gave *msp-1* an expected heterozygosity (He) of 0.915, *msp-2* an He of 0.963, and *glurp* an He of 0.956 (see Data Set S1 in the supplemental material for full data). It was assumed that the genotypes of initial clones were independent of each other and independent of the genotypes of reinfections (i.e., it was assumed there is no local genetic structuring of the malaria population). Note that alleles at *msp-1* and *msp-2* exist in our distributions as members of three or two distinct families, respectively.

Once the patient parasite dynamics were modeled (as described above), and genetic profiles at the three loci were assigned, our models followed the same process as *in vivo* trials. Blood samples were taken from each patient immediately prior to treatment (the initial or “baseline” sample) and at predetermined days during the follow-up period. Samples were screened for the presence of Plasmodium falciparum by light microscopy, and any detected infection was labeled a “recurrence.” We simulated the genotyping that would be used *in vivo* to obtain the genetic profiles for all initial and recurrent infections. Recovering the genetic signal that would be observed at time of treatment and at any recurrence reflected the technical limitations of acquiring blood samples and genotyping as follows:

• A “sampling limit” exists; a finite amount of blood is used for genotyping. A parasite clone (and consequently, its alleles) would not be detected if its density were so low that no parasites are included in the blood sample analyzed. Thus, the density and volume of the processed blood sample define the limit of detection. Obviously, this sampling limit differs between methods and laboratories. Typically, the equivalent of 1 μl of whole blood is introduced into PCR. An assumption there is 5 liters of blood in the human body gives a total of 5 × 10^6^ μl of blood. For a clone to be detected, a minimum of 1 parasite (which carries a single DNA template) would need to be present in 1 μl of blood, so there would need to be present at least 5 × 10^6^ parasites representing a given clone for that clone to be physically sampled in the genotyping process. We also needed to allow for the fact that suboptimal storage conditions (such as temperature) frequently occur in the field and will lead to DNA template breakages, and there is periodical absence from the peripheral blood of sequestered parasites. Consequently, the limit of detection will be much higher than 1 parasite per μl of blood. We therefore assumed 10 to 20 parasites per μl would be required to reliably contribute a genetic signal and ensure its detection, corresponding to a total parasitemia of 5 × 10^7^ to 5 × 10^8^; we selected the upper limit (i.e., 10^8^) to ensure reliable detection of that clone and because it is consistent with the microscopy detection limit.
• The magnitude of the genetic signal that will be produced by each malaria allele in the blood sample was proportional to the number of parasites carrying that allele.
• An inherent feature of PCR is “template competition”: i.e., the relative detectability of alleles at each marker depended on their length, with shorter alleles being more detectable due to their being better amplified in the PCR process (11). A linear relationship between allele length and relative detectability was assumed. This was done for simplicity, but other relationships (for example, log-linear) could also be investigated. The shortest allele in each case was assumed to have a relative detectability of 1, while the longest had a relative detectability of 0.001: i.e., we assumed the shortest allele generated a thousand times the genetic signal of the longest. This number is based on calculations from reference 11. Families within *msp-1* and *msp-2* were assumed to be amplified by separate reactions (i.e. are not multiplexed), so the effect only occurred between alleles within the same families. (*glurp* does not have families, so the effect applied to all alleles.) The sensitivity of the results to this relative detectability was tested by shortening it to 0.1 (supplemental material); we later show that it does not affect our results.

The strength of the genetic signal contributed by an allele in a given blood sample was therefore the product of two factors: the number of parasites carrying the allele × the detectability of the allele. Note that genotyping detects alleles, not parasites. Hence, if two (or more) clones within the infection shared the same allele, the signal for that allele was based on the total number of parasites in the two (or more) clones. The final step is to recognize that, in practice, if one allele makes up a large proportion of the genetic signal, then the smaller signals from “minority” alleles would be rejected as background “noise.” We assumed this threshold to be 25% (i.e., that signals from alleles that were less than 25% of the highest allelic signal are rejected as “noise”), although we test other values of this parameter (supplemental material).

We do not explicitly incorporate the effect of malaria sequestration in our simulations. Sequestered stages are not detectable in blood, so if a malaria clone is asynchronous in its 48 h of development, its detectability will differ over consecutive days (31, 32)—hence the observation that sampling blood from a patient on two consecutive days greatly improves the genetic detectability of clones in the patient (33, 34). WHO recommends single-day sampling: presumably for logistical reasons and because of ethical considerations to treat infections as soon as possible. We do not wish to enter the debate about the practicality versus desirability of single- or consecutive-day sampling but simply note that our results apply to both methodologies. The effect of consecutive-day sampling is to improve genetic detectability of clones, and sensitivity analysis of our detection limit (see Fig. S11 in the supplemental material) shows that improved detectability does not qualitatively affect our conclusions.

### Classifying patients according to therapeutic outcome in trials.

For analysis of parasitemia during patient follow-up and, if required, application of molecular correction algorithms to recurrent infections, four molecular correction algorithms (and a non-PCR-corrected “algorithm”) were investigated: (i) the current WHO/MMV algorithm (3), (ii) a no-*glurp* algorithm that only considers *msp-1* and *msp-2*, (iii) a ≥2/3 markers algorithm that considers *msp-1*, *msp-2*, and *glurp* but requires matching alleles at only two markers to classify a recrudescence, and (iv) an allelic family switch algorithm that considers only *msp-1* and *msp-2* and requires a family shift to classify a recrudescence if the markers are discordant (i.e., one has shared alleles between the initial and recurrent infections and one does not). Full details of these algorithms are presented in Table 1; they enabled each patient to be classified across four groups as would occur in a real trial:

(i) An early treatment failure (ETF) if a recurrence occurs on or before day 7. Note that all such recurrences are regarded as drug failures, and molecular correction is not required. In our simulations, on day 3, if total parasitemia exceeded 108 but was <25% of the total parasitaemia of the initial sample, the patient continued in the trial per the WHO protocol (consequently, no genotype was taken of the day 3 sample and no classification was made). If parasites were present at >25% of initial parasitemia, that patient was classified as an early treatment failure, consistent with the WHO procedure (2). For the purposes of estimating failure rates in this methodology, we do not distinguish between early treatment failure and recrudescence as both are indicative of drug failure.
(ii) A drug failure if a recurrence was classified as such by a PCR correction algorithm in Table 1.
(iii) A reinfection if a recurrence was classified as such by a PCR correction algorithm in Table 1.
(iv) “Cleared”: i.e. no recurrent parasitemia was detected during follow-up. In these cases, the drug was assumed to have successfully killed all parasites present at time of treatment.

A key objective of this paper was to investigate how well the classification algorithms applied to recurrent infections (Table 1) recovered the true status of recurrent infections. We therefore defined the latter according to parasitemia data from the PK/PD model (Fig. 1).

• True recrudescence was defined as a recurrent infection that contained at least 10^8^ parasites from a clone present at time of treatment. (This patient is, by definition, a drug failure.) This included patients who have a “mixed” infection on the day of recurrence (i.e., possessed malaria clones that survived treatment plus reinfection clones that were acquired during follow up, providing the former exceed 10^8^). Note that all clones contributed to the genetic signal of the recurrence as described above.
• True reinfection was defined as a recurrent infection whose blood sample contained only parasites from a clone or clones that were reinfections. (Note that such patients may harbor parasites from original clones if these clones were subpatent [i.e., less than 10^8^ parasites].)

It was possible that recrudescent clones may not have reached microscopically detectable levels (i.e., parasite numbers are <10^8^) on the final day of follow-up; such patients would be classified as “cleared” *in vivo* and thus a treatment success. However, simulated data have confirmed that it is possible for some patients to still harbor parasites below the detection level at the end of follow-up (13). Our modeling approach classifies these patients as drug failures.

Note there are only a finite number of alleles at each locus, and thus, two distinct clones of malaria may have had identical alleles at one or more markers purely by chance. It followed that reinfections and recrudescences could share alleles, so misclassification of reinfection as recrudescence was possible.

### Estimating drug failure rates in the simulated trials.

The model was run for a cohort of 5,000 patients (although any number can be simulated). This is an unrealistically high number for an *in vivo* clinical trial but is ideal for our purposes. A true drug failure rate of 10 to 12% provided a large number of recurrences (the exact number varying depending on the ACT, FOI, and length of follow-up) that we can test against the various classification algorithms and reduces the uncertainty around results.

The four patient outcomes described above were used to calculate the estimated drug failure rate, *F̂*, in the same manner as outcomes reported *in vivo*. It was assumed, for simplicity, that no patients were lost to follow-up or removed from the trial for any reason other than recurrent parasitemia. There were three methods for calculating failure rates, which differed in how they processed patients with recurrent parasitemia that had been classified as reinfections, noting that all patients with recurrent parasitemia would, *in vivo*, be treated again with another antimalarial (for ethical reasons) and removed from the trial. The three methods were a non-PCR-corrected failure rate, a “per protocol” failure rate, and a failure rate obtained using survival analysis. The latter two methods are recommended by the WHO to analyze antimalarial drug trials (2, 3). Technically, they were calculated as follows using the following nomenclature:

- *C_o_* was the number of patients who cleared infection.
- *nI_o_* was the number of patients whose recurrent infections were classified as reinfections.
- *F̂* was the estimated drug failure rate.
- *N* was the total number of patients.

(i) The non-PCR-corrected failure rate was obtained by considering all patients with recurrent infections as patients who had failed drug treatment. This method did not require distinguishing between reinfections and recrudescent infections. The estimated failure rate, *F̂*, could then be estimated as

(1) F^=1−CoN

(ii) The per protocol method, recommended by WHO (2, 3, 5), simply removed patients who were classified as reinfections from the total number of observations:

(2) F^=1−CoN−(nIo)

(iii) Survival analysis, as recommended by WHO (3), used the survivor function from a Kaplan-Meier plot on the final day of follow-up, right-censoring reinfections.

The Kaplan-Meier (KM) estimator of survivorship at time *t*, *Ŝ*(*t*), was obtained as

(3) S^(t)=∏ti≤tni−dini

where *t* was a vector of all time points (i.e., days of follow-up in which an event occurred in the study population), *ni* was the number of individuals at time *ti* who remained uninfected, and *di* was the number of events (drug failures in this case) that occurred at time point *ti*. Plainly, what this method did was calculate the proportion of patients who remained free of recrudescence between consecutive days of follow-up, then multiply all these time periods to obtain the overall probability of “surviving” recrudescence free over the whole follow-up period. The advantage was that even those patients who are “censored” (by acquiring a reinfection and leaving the study) will still contribute to the analysis through their inclusion prior to their removal.

The estimator at the final time point (i.e., the last day of follow-up) was the probability that their treatment was considered a “success” at the end of the trial. Consequently, its complement gave the probability that a given individual will fail treatment:

(4) F^=1−S^(t)

The final methodological step was to interrogate the modeled data to determine the “true failure rate”—i.e., the drug failure rate calculated directly from the parasitemia of each patient (thus, not dependent on genotyped data). For each patient in the simulation, an outcome on the final day of follow-up was determined: if, on the final day, the patient had any parasites from any initial clones (i.e., even a single parasite), the patient was denoted as a drug failure. If no parasites had survived from the initial clones present at treatment, that patient was denoted as a treatment success.

The true failure rate, *F*, for the patient population was then calculated as

(5) $$F=\frac{f}{N}$$

where *f* was the number of drug failures on the final day of follow-up and *N* was the total number of patients.

This was the “gold standard” metric and cannot be obtained *in vivo*. It was compared to the estimated failure rates obtained from modeling the clinical trial and molecular correction process and allowed us to quantify the accuracy of different methods (i.e., their ability to recover the true failure rate).

### Reanalysis of existing *in vivo* data.

Clinical data were obtained from Rwanda (a relatively high-transmission area) across 6 sites between 2013 and 2015, where patients were treated with either AR-LF or DHA-PPQ and genotyped at *msp-1*, *msp-2*, and *glurp*. In patients treated with AR-LF, 137 recurrences were observed, of which 110 could be classified as either a reinfection or a recrudescence. (It was not possible to classify 27 patients because they had incomplete genetic data.) In patients treated with DHA-PPQ, 48 recurrences were observed, of which 43 could be classified as either a reinfection or a recrudescence. (It was not possible to classify 5 patients because they had incomplete genetic data.) These data were initially presented internally to the National Malaria Control Program in Rwanda (unpublished data).

Clinical data from Cambodia (a relatively low-transmission area) were obtained from 6 sites between 2014 and 2016. Patients were treated with either artesunate plus amodiaquine (AS-AQ), artesunate plus pyronaridine (AS-PYN), or DHA-PPQ and genotyped at *msp-1*, *msp-2*, and *glurp*. In patients treated with AS-AQ, 12 recurrences were observed, of which 5 could be classified as reinfection or recrudescence. (Seven patients had incomplete genetic data.) In patients treated with AS-PYN, 14 recurrences were observed, of which 12 could be classified as reinfection or recrudescence (2 had incomplete genetic data). In patients treated with DHA-PPQ, 67 recurrences were observed, of which 48 could be classified as reinfection or recrudescence. (Nineteen had incomplete genetic data.) These data were initially presented internally to the National Malaria Control Program in Cambodia. A description of the AS-PYN trials has already been published (35).

For all data, the genetic signals (i.e., the *msp-1*, *msp-2*, and *glurp* alleles at the initial sample and any recurrent sample) were reinterpreted using the novel molecular correction algorithms described in Table 1 to investigate how varying the molecular correction algorithm changed the classification (as reinfection or recrudescence) of patients and, consequently, failure rate estimates.

### Data availability.

The R code used to generate the results describe herein is available from the authors. The reanalyzed trial data sets are likewise available from the authors.

## Supplementary Material

Supplemental file 1

Supplemental file 2
