# Supplementary material for: Improving Methods for Analyzing Antimalarial Drug Efficacy Trials: Molecular Correction Based on Length-Polymorphic Markers msp-1, msp-2, and glurp
Source: Antimicrob Agents Chemother. 2019 Aug 23;63(9):e00590-19. doi: 10.1128/AAC.00590-19 (PMC6709465; doi:10.1128/AAC.00590-19)
Supplement: Supplemental file 2 [file AAC.00590-19-s0002.pdf]

Supplemental Material

Additional Methodology

Simulating parasite dynamics post-treatment *in silico* with a pharmacokinetic pharmacodynamic (PK/PD) model – choice of parameters and validity of the results with use of other parameters.

These relate to drug concentration (blue line in Figure 1) and changes in parasite number over time (red, green, grey and orange lines in Figure 1).

Pharmacokinetics (PK) determines a drug's concentration-time profile (solid blue line in Figure 1).

Pharmacodynamics (PD) describes the sensitivity of the parasites to the drug and determines how the number of parasites changes as a function of drug concentration within a patient over time (dotted lines in Figure 1

Three artemisinin-based combination therapies (ACTs) were investigated in this study: Dihydroarteminisin-Piperaquine (DHA-PPQ), Artemether-Lumefantrine (AR-LF) and Artesunate-Mefloquine (AS-MQ). The mechanistic simulation of these drugs has been defined, calibrated and validated extensively in our previous work e.g. (1-5). The parameterization of these drugs in these simulations is provided in Table S1. Patient weight in the simulations (involved in the calculation of PK parameters for PPQ) was drawn from a uniform distribution between 45-75 kg. PK parameters for all drugs vary extensively in the literature, a fact that is not surprising given that studies are drawn from a variety of patient populations (see (5) for examples). We do not try to replicate any *given* population (and thus, their PK values) – rather we choose a mean value that reflects a large proportion of studies and choose a coefficient of variation (CV) for each parameter that is sufficiently large to encompass a sensible range of parameters (and consequently, drug concentration over time profiles), in order to model a "general" patient population over our large trial size (5,000 patients). These values are shown in full in Table S1 .

There is evidence of DHA-PPQ having high estimated failure rates *in vivo*, and well-documented PD parameterization for this ACT as it fails (Saunders and colleagues (6) estimated PPQ IC50 had increased to 23.9ng/ml in recrudescent infections as resistance spread; this is equivalent to 0.024 mg/L which we round to 0.02mg/L in our calibration, see Table S1). Consequently, simulating failing DHA-PPQ using *in vivo* data to calibrate the model was possible and produced a 12% true failure rate with the MOI from Tanzania described in methods. There are field data allowing calibration of PK/PD parameters for non-failing AR-LF and AS-MQ (the *in vivo* parameters given in Table S1 that produce a 0.05% and 2% true failure rate respectively. Ideally, we would use field PK/PD calibrations for each ACT obtained from locations where the drug was failing but failing AR-LF and AS-MQ have not been observed in any known PK/PD studies. To avoid drawing conclusions based on analysis of a single failing drug (i.e. DHA-PPQ), we produced 'failing' calibrations of AR-LF and AS-MQ by artificially increasing the parasites' mean IC50 values (Table S1) until the simulated drug failure rates reached 9% and 10% respectively. This reflected plausible future scenarios that may occur as resistance arises to these drugs. We inflated failure rates to around 10% because this is the critical point at which WHO recommend a drug be withdrawn from front-line usage (7) so it was important to evaluate the accuracy of the various methods around this critical point. LF and MQ have very different durations of protection post-treatment so comparison of the three failing drugs allowed us to investigate different durations of follow-up post treatment. Note that we only changed the IC50 of the partner drug, to get high levels of failure for AR-LF and AS-MQ and did not alter sensitivity to the artemisinin component; drug failures still must survive artemisinin killing thus these partner-drug IC50 values may be higher than would be expected for monotherapy resistance.

The parasite dynamics for DHA-PPQ in the main text were created using a one-compartment PK model for DHA and a two-compartment PK model for PPQ (parameters in Table S1). Reported PK values for PPQ vary widely in the literature (see (5) for examples though note this is obviously unsurprising as PK values are drawn from studies of different populations) and PPQ can be calibrated in a one, two or three compartment model. To show our results are consistent across multiple PK calibrations and for

completeness, we generated parasite dynamics using a three-compartment model described in (8) with PK parameters based on the mean values reported in table 2 of (8) and in our Table S2. Comparison of drug concentration over time for a single patient with this three compartment calibration and the two compartment calibration (i.e., mean parameters shown in Table S1 against mean parameters in table 2 of (8)) is shown in Figure S1.

Note that we did not incorporate the error model or covariate effects described in (8); we were not trying to re-create their patient population (which is a mix of pregnant and non-pregnant women), rather we were trying to create parasite dynamics for a general patient population under the assumption of a three compartment PPQ model and so use the mean values for PK parameters in (8) as a base. As with the parameterizations for two-compartment PPQ and the other drugs, we then used relatively large coefficients of variation across 5,000 patients (Table S2).

The principal difference between the parasite dynamics generated with these assumptions is that the three-compartment model is slightly more prophylactic and has a greater total area under the drug kill curve; consequently, true failure rate is slightly lower, and a smaller number of reinfections become patent. However, failure rate estimates obtained using each algorithm are not significantly different between the two compartment and three compartment models, and we later show our results (the relative performance of molecular correction algorithms) are qualitatively the same with both model calibrations. We are not attempting to comment, here, on whether DHA-PPQ is best represented by a two or three compartment model or its exact parameterization. Furthermore, we are not trying to reproduce the PK of a given population of patients reported anywhere in the literature, but rather produce a general population of patients with parasite dynamics post-treatment we can use to analyse molecular correction algorithms (which we achieve by setting the CV on our parameters such that we cover a wide range). We simply confirm and stress the consistency of the molecular correction algorithms across both parameterizations, suggesting that, regardless of the

number of PPQ PK compartments included, our conclusions regarding the accuracy of these molecular correction algorithms to estimate treatment failure rates are robust.

We did not have access to validated PK/PD models for other common partner drugs i.e. Amodiaquine (AQ), sulfadoxine/pyrimethamine (SP) and pyronaridine. Both the parent form and metabolite of AQ have antimalarial activity, they are both best described with multiple PK compartment models and both are eliminated independently (e.g. (9)): we were unable to obtain robust PK/PD models (10). SP exhibits strong synergy between the sulfadoxine and pyrimethamine components which again makes it difficult to get a robust PK/PD model (11). Finally, pyronaridine is so new that we have not yet had the time or resources to attempt a PK/PD model of this treatment. However, the three drugs that we can investigate have different periods of chemoprophylaxis post treatment and are likely good guides for other drugs: specific calibrations of PK/PD models cannot affect the genetic profiles obtained prior to treatment and there seems no obvious reason why they would alter the genetic profiles of recurrent infections. so we argue that the three examples are sufficient to generate robust results for the analysis of *msp*-1, *msp*-2 and *glurp* markers.

Force of Infection: the rate of emergence of reinfections during the follow-up period

Our selection of FOI values was based on the following literature: Data from northern Ghana indicates that the average number of reinfections per patient per year was 16, and similar estimates can be obtained from efficacy data of effective ACTs (see supplementary material of (2)). Mueller et al. (12) obtain estimates of between 3 and 9 reinfections emerging per year with an average of 5.9 in Papa New Guinea. Additional work suggests the FOI in Ghana is highly seasonal with estimates ranging from 44 in the high transmission season to 7 in the low transmission season (13); any yearly average (such as assumed in this manuscript) will fail to capture the nuances of seasonal transmission. Smith et al. (14) explicitly modelled the relationship between EIR and FOI. It is technical, but some illustrative data are summarised in their Figure 2: Incidence during a 2 week period at annual EIRs of 36.5 (moderate

transmission) and 365 (high transmission) were roughly 0.2 and 0.4 respectively implying annual FOI estimates of 0.2 x26=5.2 and 0.4x26= 10.4 respectively. These may be slight under-estimates because this simple calculation assumed that more than one infection could not become established in a 2 week period but serves as general illustrations of the relationship between EIR and FOI.

Additional Results

Misclassification of recurrent infections for DHA-PPQ with varying FOI levels

Figure 3 (main text) shows the misclassification of recurrent infections (recrudescence classified as reinfection and vice versa) for an FOI of 8. Figure S2 shows the same plot for an FOI of 2, 8, and 16. It shows that the number of recrudescence misclassified as reinfection is stable as FOI increased for all algorithms. Furthermore, it shows that increased FOI had nearly no impact on the number of reinfections being misclassified for the "WHO/MMV" algorithm (which correctly classified all reinfections), and a very minor impact for the "no glurp" algorithm. For the "≥ 2/3 markers" and "allelic family switch" algorithm, this figure demonstrates that increased FOI led to greatly increased numbers of reinfections being misclassified as recrudescence. The *proportion* of reinfections misclassified was stable as FOI increased, but the greater total number of misclassifications produced the increased failure rates seen with these algorithms in Figure 4 (main text).

Results for failing DHA-PPQ with a three-compartment calibration

We generated parasite dynamics for each patient using a three-compartment model calibration for DHA-PPQ (rather than the two-compartment calibration shown in the main text), i.e. Table S2 . The results are shown in Figure S3. The qualitative patterns were the same as for the two-compartment model, i.e., that "WHO/MMV" algorithm produced the lowest failure rate estimate, then "no glurp," then "≥ 2/3 markers", then "allelic family switch" (at most FOI, "≥ 2/3 markers" produced a slightly higher failure rate estimate at 0-2 FOI). True failure rate was slightly lower for the three compartment model (10% vs 12%). Failure rate estimates with all algorithms, given the same length of follow-up and

FOI, were lower with a three compartment model (likely due to its longer prophylactic period, see Figure S1). Thus, while the "≥ 2/3 markers" algorithm produced an accurate estimate at most FOI (though "no correction" is better with an FOI of 0) with a 42 day follow-up for a two compartment model, assuming a three compartment model of DHA-PPQ showed that "≥ 2/3 markers" produced accurate failure rate estimates but with a follow-up period of 63 days; an intuitive result given the longer prophylactic period.

Results for failing Artemether-Lumefantrine (AR-LF) and failing Artesunate-Mefloquine (AS-MQ) compared with failing Dihydroarteminisin-Piperaquine (DHA-PPQ): The impact of different correction algorithms on estimated drug failure rates, and appropriate durations of trial follow-up.

We investigated failing AR-LF and failing AS-MQ to confirm that the same patterns were observed as for failing DHA-PPQ. The results are discussed here to save space and to maintain focus on the key points in the main manuscript.

Failure rate estimates for DHA-PPQ with 28, 42 and 63 day follow-up periods are shown in Figure 4 Failure rate estimates increased as follow-up length increases because a) more true recrudescences became patent and b) more reinfections became patent that may be misclassified as recrudescent (see discussion in main manuscript Figure 4). Consequently, our results (main text) suggested that use of the "≥ 2/3 markers" algorithm and a 42-day follow-up was the most appropriate option for DHA-PPQ trials.

Failure rate estimates for failing AR-LF for 21-day and 28-day follow-up lengths are presented in Figure S4 . The true failure rate of AR-LF in these simulations was 0.918 (9%). The same pattern was observed as for DHA-PPQ: The non-PCR corrected algorithm over-estimated the failure rate at any FOI higher

than 1, and severely overestimated failure rates at high FOI; the "WHO/MMV" algorithm and the "no glurp" algorithm slightly under-estimated the failure rate across all levels of FOI. Use of a 21-day follow-up period led to both the "allelic family switch" algorithm and the "≥ 2/3 markers" algorithm under-estimating the failure rate, only at a high FOI of 13 did the allelic family switch algorithm accurately recover the true failure rate. Use of a 28-day follow-up period produced more accurate failure rate estimates: The "≥ 2/3 markers" algorithm accurately recovered the true failure rate between an FOI of 5-16, with both the "≥ 2/3 markers" algorithm and the "allelic family" switch algorithm under-estimating the failure rate slightly at lower FOI. These results combined with the true classifications of recurrent infections recrudescences and reinfections (Figure S5) suggested a 28-day follow-up period led to more accurate failure rate estimates.

Failure rate estimates for failing AS-MQ for a 42, 49 and 63-day follow-up length are presented in Figure S6 . The true failure rate of AS-MQ in these simulations was 0.1032(10%). With a 42-day follow-up period (Figure S6 (A)), the "≥ 2/3 markers algorithm" under-estimated the true simulated failure rate at all FOI settings – the "allelic family switch" and "≥ 2/3 markers" algorithm were close in value up to an FOI of 9-10. As with DHA-PPQ and AR-LF, the "WHO/MMV" and "no glurp" algorithms under-estimated the failure rate consistently and using no PCR correction generated a large over-estimate of the true failure rate. We simulated a novel follow-up length of 49 days (Figure S6 (B)) under which the "≥ 2/3 markers" algorithm produced a more accurate failure rate estimate than a 42-day follow-up at all FOI levels. With a 63-day follow-up period (Figure S6 (C)), the "allelic family switch" algorithm over-estimated the true failure rate from an FOI of 4 and upwards. The "≥ 2/3 markers" algorithm over-estimated from an FOI of 8 and up, but only by a small amount. AS-MQ is more prophylactic than DHA-PPQ and AR-LF: Given the same period of follow-up, fewer reinfections became patent, and recrudescences occurred later in the follow-up period (Figure S7). As such, it was unsurprising that a longer period of follow-up led to more accurate failure rate estimates. Using the "≥ 2/3 markers" algorithm and assuming an FOI of <8, a 63-day follow-up period resulted in a more accurate estimate

than the 42 and 49-day follow-up lengths, but the differences in estimates between 49 and 63 days were small and the operational, logistical advantages of a 49-day trial over a 63-day trial are likely to be substantial. Furthermore, with an FOI of ≥8, a shorter follow-up (49 days) produced a more accurate failure rate estimate with the "≥ 2/3 markers" algorithm – a 63 day follow-up period over-estimated the true failure rate slightly with higher transmission intensity using this algorithm.

In summary, the results for the three failing drug calibrations differed slightly quantitatively, but the same qualitive patterns occurred i.e. the "WHO/MMV" method returned large underestimates (around two thirds the true value) of failure rates, while the "≥ 2/3 markers" algorithm produced consistently more accurate estimates, with some dependency on transmission intensity (quantified by FOI). Increased length of follow-up increased failure rate estimates, as for DHA-PPQ, though due to the different prophylactic profiles of the drugs the accuracy of failure rate estimates at a given length of follow-up differed.

Results for non-failing (effective) AR-LF and AS-MQ

The simulations were run for the non-failing (i.e. effective drug) PK/PD calibrations for AR-LF (Figure S8) and AS-MQ (Figure S9), which had true failure rates of 0.0046 (0.5%) and 0.0208 (2%) respectively. This was to investigate whether the new algorithms could incorrectly classify effective drugs as failing. Crucially, the under-estimate associated with of the "≥ 2/3 markers" algorithm was so small in terms of absolute value that the use of the algorithm can be recommended without concern for over-estimating the failure rate of effective drugs i.e. there is no danger of an effective drug being misclassified as failing. These results do highlight the dangers of not using a molecular correction: The non-PCR-corrected algorithm generated estimated failure rates >10% in areas of high FOI when using long durations of follow-up. The WHO recommend that drugs be replaced when failure rates exceed 10% (7), so not using molecular correction could lead to unwarranted policy change.

Sensitivity analysis of multiplicity of infection (MOI), relative detectability of alleles and the minority allele detection threshold

The results presented in the main text all assumed MOI at time of treatment is representative of high transmission i.e. using Tanzanian data (see MOI in main text). We did this because high MOI makes detection of recrudescent alleles more difficult (due to the issues described in our methods section with detection of minority alleles) so represents a "worst case" scenario. There is a likely mismatch for areas of low transmission which have lower MOI at treatment, but we used high MOI across all transmission intensities (quantified by FOI) for the following reasons:

- Keeping the same MOI across all transmission intensities allowed a direct comparison of molecular correction algorithms (e.g. Figure 2, Figure 4)
- This assumption of high MOI at treatment is conservative (i.e. "worst case" scenarios) for low transmission areas because we show that there is little operational difference between the algorithms even if initial MOI is high; it is therefore a robust conclusion that algorithm choice is not important in these areas because if MOI at treatment is lower, then there will be even less difference between the algorithms (as illustrated by the Cambodian field data that showed negligible differences).
- High MOI at time of treatment can occur even in low transmission areas if people immigrate from areas of higher transmission or have acquired sufficient protective immunity that several clones may co-circulate asymptomatically before the patient falls ill. More plausibly, this scenario may arise in areas of seasonally intense transmission where MOI at time of treatment is high, but trials are conducted during the low-transmission season to reduce the impact of reinfections.

We checked the impact of reduced MOI. Analysis of simulated data for DHA-PPQ with a 42-day follow-up and a low MOI setting (the distribution obtained from PNG; see methods) is shown in Figure S10. First note that the true failure rate was slightly lower than that obtained in a high MOI setting (Figure 4) because patients harboured fewer clones at time of treatment which made their infection easier to clear. Reducing the MOI to reflect a low-transmission setting reduced the difference between algorithms. Overall, the results were consistent with those obtained from a high MOI setting i.e. the "allelic family switch" algorithm produced an accurate failure rate estimate at an FOI of 4 and below, and the "≥ 2/3 markers" algorithm produced the most accurate failure rate estimate at all higher FOI.

The relative detectability of the longest allele to the shortest allele was altered from 0.001:1 to 0.1:1. The results are shown in Figure S11. Failure rate estimates obtained using this altered relative detectability are nearly identical to those obtained with the relative detectability of 0.001:1 used elsewhere in this manuscript (i.e. Figure 2 of main text).

The threshold at which minority genotyping signals are discounted as "noise" and disregarded was varied from 0.3 to 0.05. Analysis of simulated data for DHA-PPQ with a 42-day follow-up under these conditions is shown in Figure S12. The failure rate estimate produced by each algorithm increased as the threshold decreased. At the lower threshold of 0.05 the "no glurp" algorithm (rather than the "≥ 2/3 markers" algorithm) produced the most accurate failure rate estimate from an FOI of 6 and higher. A minority detection threshold of 0.05 is unrealistic because large amounts of experimental/laboratory noise would be included in the signal, so this threshold could not be used in practice. The threshold was changed to 0.2 (a more realistic value) in Figure S13. Under this assumption the "≥ 2/3 markers" algorithm produced the most accurate failure rate estimate, robust across all FOI levels, the same as when the minority detection threshold is set to 0.3.

Per protocol vs survival analysis for using the molecular correction data to obtain estimated failure rates.

WHO guidelines (7) recommend two methods for statistical analysis of molecular-corrected data: Survival analysis and per-protocol analysis. The results presented in the main manuscript and this supplemental material for DHA-PPQ, AR-LF, and AS-MQ are failure rate estimates obtained using survival analysis. The same models were analysed to obtain failure rate estimates calculated using per-protocol method Figure S14 to Figure S16). Comparison of these results showed that the per protocol method generates slightly higher estimated failure rates than survival analysis. The differences were dependant on the FOI level and duration of follow-up – the more reinfections that become patent over the course of follow-up (as occurs with higher FOI and longer follow-up), the greater this difference. With a 63-day follow-up and an FOI of 16 the failure rate estimate obtained for DHA-PPQ with the per-protocol method was nearly 30%, compared to the estimate with survival analysis of 15%. The reason is a "denominator effect". The per-protocol analysis simply removes all patients identified with reinfections from the analysis. Take the example where 20 of 200 patients are drug failures, giving a true underlying failure rate of 20/(20+180)=10%. If, for example, 50 of the 180 cured patients had reinfections and were removed from the analysis then the estimated per-protocol failure rate would rise to 20/(20+130)= 13% and if 100 of the cured patients had reinfections then failure rate would further increase to 20/(20+80)=20%. This example is somewhat artificial because reinfections will also occur in the recrudescence group and if they occur first, a later recrudescence could be masked, but it does serve to illustrate this denominator effect. It is important to appreciate that use of the per-protocol method with the newly proposed "≥ 2/3 markers" algorithm (which generally produced more accurate failure rate estimates with appropriate follow-up length, see main text) will result in an over-estimate of failure rate. A detailed discussion of statistical analysis of malaria drug trials can be found

elsewhere (15) but here we emphasise that reporting the failure rate estimate obtained through survival analysis is essential with the use of this new algorithm.

Additional discussion

Alternative markers for molecular correction

We focused on the currently recommend WHO genetic markers and methods in the main text. Optimising their use is the current priority but looking forward, there are alterative methodologies and markers than may be used and which may be superior. These markers and methods will be addressed in future studies but, for the record, the three main alternative markers are as follows.

- Amplicon sequencing of marker loci (16). Its main advantage over capillary electrophoresis of *msp-1*, *msp-2* and *glurp* is that deep sequencing allows very sensitive detection of minor clones. Minority clones that had a frequency >1.0% of all reads were consistently detected (16). We anticipate that this sensitivity will favour a “WHO/MMV”-type algorithm (i.e. a recrudescence should share alleles at all amplicons when comparing initial and recurrent samples) as the use of amplicon sequencing should improve detection of minor clones in the initial sample (reducing the number of recrudescent clones being misclassified as reinfection) and will be better able to detect recrudescent clones in mixed infection recurrences.
- Microsatellite loci have already been used in antimalarial efficacy studies (17, 18). Microsatellites are similar to the *msp*-1, *msp*-2, *glurp* markers as their sensitivity to detect minor clones is relatively weak (in particular the presence of stutter-bands require a stringent cut-off) but more loci are often genotyped (Plucinski et al (19) used 8 microsatellites), which means there are a greater number of potential algorithms that may be constructed to

distinguish recrudescences from reinfections. In addition, there is a Bayesian analysis method for these markers which may improve their role in molecular correction ((17))

- Finally, SNP barcodes may be used as genetic markers.

The intention here is not to provide an exhaustive description of alternative markers but to emphasise that it is straightforward to assign such genotypes to our simulated patients in the same way that we assigned the *msp-1*, *msp-2* and *glurp* genotypes, and test various classification algorithms based on such loci. Finally, we note that existing algorithms simply classify recurrent infections as either reinfections or recrudescences and do not account for any degree of uncertainly in these classifications; for example, although we recommend the "≥ 2/3 markers" algorithm, we may be more confident that a recurrent infection is a drug failure if it shares identical alleles at all 3 loci than if it shares alleles at only 2 loci. A natural way of incorporating such uncertainty is to use Bayesian methods and a recent paper has identified such a technique (19); we will evaluate this method in our future work. In short, validated simulations of drug treatment and the consequent post-treatment parasite dynamics provide an ideal resource to investigate many issues surrounding the design, implementation and analysis of clinic trials and we commend their use as a test platform to other interested parties working to improve the design and analysis of malaria drug clinical trials.

Drug parameter	Dihydroarteminisin-Piperaquine (two-compartment model)		Artesunate-Mefloquine			Artemether-Lumefantrine		
	DHA	PPQ	AS	DHA	MQ	AR	DHA	LF
**Vd (L/kg)**	1.49 [0.48](1, 20)	173 [0.93](21)	7.1 [0.94](1)	1.49[0.48](1)	20.8[0.38](1)	46.6[0.82](20)	15[0.48](1, 20)	21[2.63](1, 20)
**Vd_1 (L/kg)**	-	443 [1.70](21)	-	-	-	-	-	-
**ka (/day)**	-	11.2 [2.17](21)	252[1.12](1)	-	-	23.98[0.68](1, 20)	-	-
**z (/day)**	-	-	30.96[0.362](1)	-	-	11.97[0.65](1, 20)	-	-
**Q_1(L/day/kg)**	-	69.7[1.01](21)	-	-	-	-	-	-
**k (/day)**	19.8[0.23](3, 20)	0.02*(21, 22)	-	25.4[0.23](1)	0.053[0.63](1)	-	44.15[0.23](1, 20)	0.16[0.05](1, 20)
**IC50 (mg/L)**	0.009 [1.17](1, 20)	0.02 [0.3](6)	0.0016[0.86](1)	0.009[1.17](1)	0.3[0.78] <0.027[0.78]> (1)	0.0023[0.79](1, 20)	0.009[1.17](1, 20)	4[1.02] <0.032(1.02)>(1, 20)

**Vmax**	27.6[0.3](1, 20)	3.45[0.3] (3)	27.6[0.3] (1)	27.6[0.3] (1)	3.45[0.3] (1)	27.6[0.3] (1, 20)	27.6[0.3] (1, 20)	3.45[0.3] (1, 20)
**n**	4[0.3](1, 3, 20)	6[0.3] (3)	4[0.3] (1, 3)	4[0.3] (1, 3)	5[0.3] (1)	4[0.3] (1, 3, 20)	4[0.3] (1, 3, 20)	4[0.3] (1, 3, 20)

Table S1 : A summary of the PKPD parameters used to generate parasite dynamics post-treatment; means with coefficient of variation (CV) in square brackets. There are two IC50 values for Lumefantrine (LF) and Mefloquine (MQ): the failing “resistant” IC50s are provided first and drug sensitive IC50 values are shown in </>. Note that IC50 values for failing LF and MQ were arbitrarily increased by us to obtain ~10% drug failure rate. We only changed the IC50 of the partner drug, so to get high levels of failure we needed to overcome the artemisinin component (whose IC50 was not changed) – thus these IC50 values will be higher than those expected for monotherapy resistance. Piperaquine (PPQ) follows a two-compartment model as described in Kay, Hodel & Hastings (21). Patient bodyweight (BW) in the simulations was drawn from a uniform distribution between 45-75 kg and is involved in the calculations for PPQ parameters (see (21, 22)). The numbers provided in brackets in the table are citations in support of the parameter values

PK/PD: Pharmacokinetic/Pharmacodynamic, BW: Patient bodyweight, DHA: Dihydroartemisinin, PPQ: Piperaquine, AS: Artesunate, MQ: Mefloquine, AR: Artemether, LF: Lumefantrine, BW: Patient bodyweight Vd: Volume of Distribution (central compartment for PPQ), Vd_1: Volume of Distributions (peripheral compartment for PPQ), ka: Absorption rate constant, z: Conversion rate of AR/AS into DHA, Q_1: Intercompartmental clearance between central and peripheral compartment (for PPQ). k: Elimination rate, IC50: Drug concentration at which 50% of maximal killing occurs, Vmax: Maximal parasite killing constant, n: slope of concentration-effect curve, - : No data / not applicable.

* elimination rate for PPQ is calculated from clearance (CL) / Vd. CL is not shown here but is 4.5 * $BW^{0.75}$ as in (22); This means that elimination rate varies with body weight (a common PK observation) so the value presented here is illustrative and represents a bodyweight of 42kg (the median bodyweight in previous studies(21, 22)).

Drug parameter	Dihydroarteminisin-Piperaquine (three compartment model)
	PPQ
**Vd (L)**	3070 [0.86](8)
**Vd_1 (L)**	4440 [1.21] (8)
**Vd_2 (L)**	31400 [0.65] (8)
**ka (/day)**	1.99 [1.08] [Table S1]
**Q_1(L/hour)**	427 [1.01](8)
**Q_2(L/hour)**	160 [0.7](8)
**k (/day)**	0.47* (8)
**IC50 (mg/L)**	0.02 [0.3](6)
**Vmax**	3.45 [0.3](3)
**n**	6 [0.3](3)

Table S2 : A summary of the PK/PD parameters used to generate parasite dynamics post-treatment PPQ with a three-compartment model (opposed to the two compartment model parameters described in Table S1 ; note that DHA parameters remain the same). PK means are derived from (8); the coefficient of variation CV; in square brackets for each parameter is added by us. PD parameters (IC50, Vmax, n) are the same as for the two-compartment model. The numbers provided in brackets in the table are citations in support of the parameter values

PK/PD: Pharmacokinetic/Pharmacodynamic, BW: Patient bodyweight, DHA: Dihydroartemisinin, PPQ: Piperaquine, Vd: Volume of Distribution (central compartment for PPQ), Vd_1: Volume of Distribution (peripheral compartment 1), Vd_2: Volume of Distribution (peripheral compartment 2), ka: Absorption rate constant, z: Conversion rate of AR/AS into DHA, Q_1: Intercompartmental clearance between central and peripheral compartment 1, Q_2: Intercompartmental clearance between central and peripheral compartment 2, k: Elimination rate, IC50: Drug concentration at which 50% of maximal killing occurs, Vmax: Maximal parasite killing constant, n: slope of concentration-effect curve, - : No data / not applicable.

* elimination rate for PPQ is calculated from clearance (CL) / Vd; CL (from (8) is 60.2 (we include a CV of 0.71 on this parameter)) so the value presented here is illustrative and represents a bodyweight of 42kg.

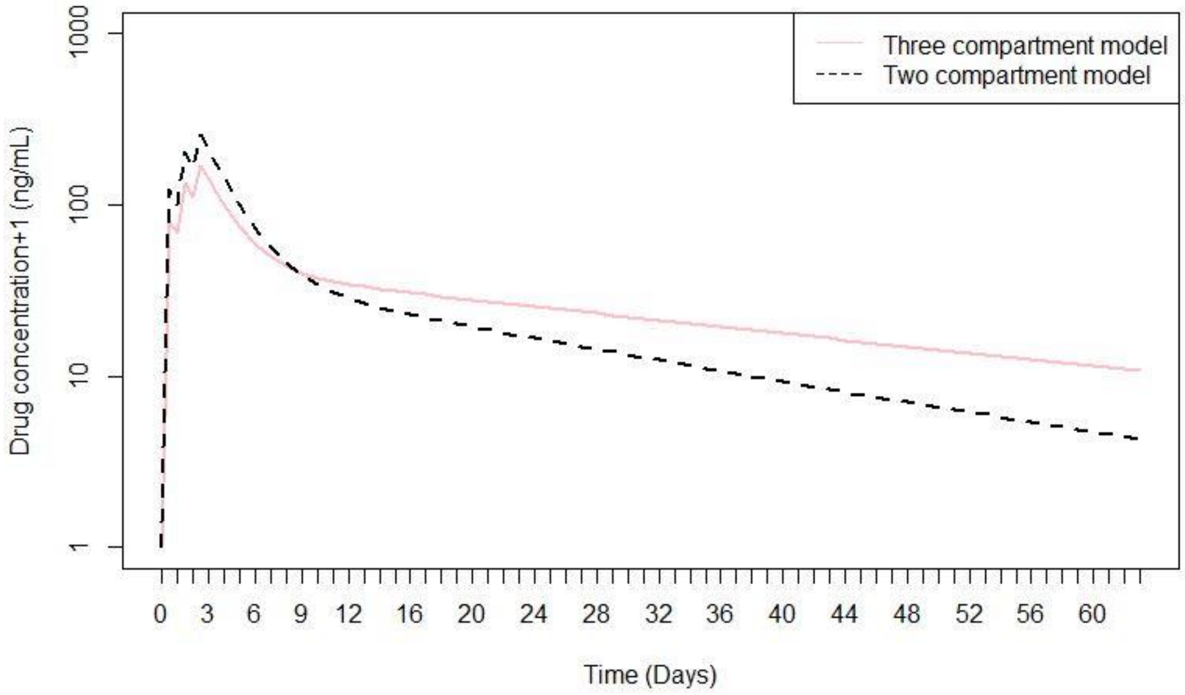

Figure S1: Comparison of drug concentration over time profiles created for a single patient with the mean parameters described in Table S1 for a two-compartment DHA-PPQ model and the mean parameters described in table 2 of (8) for a three compartment DHA-PPQ model, showing that the three compartment model produces a more prophylactic drug concentration over time profile.

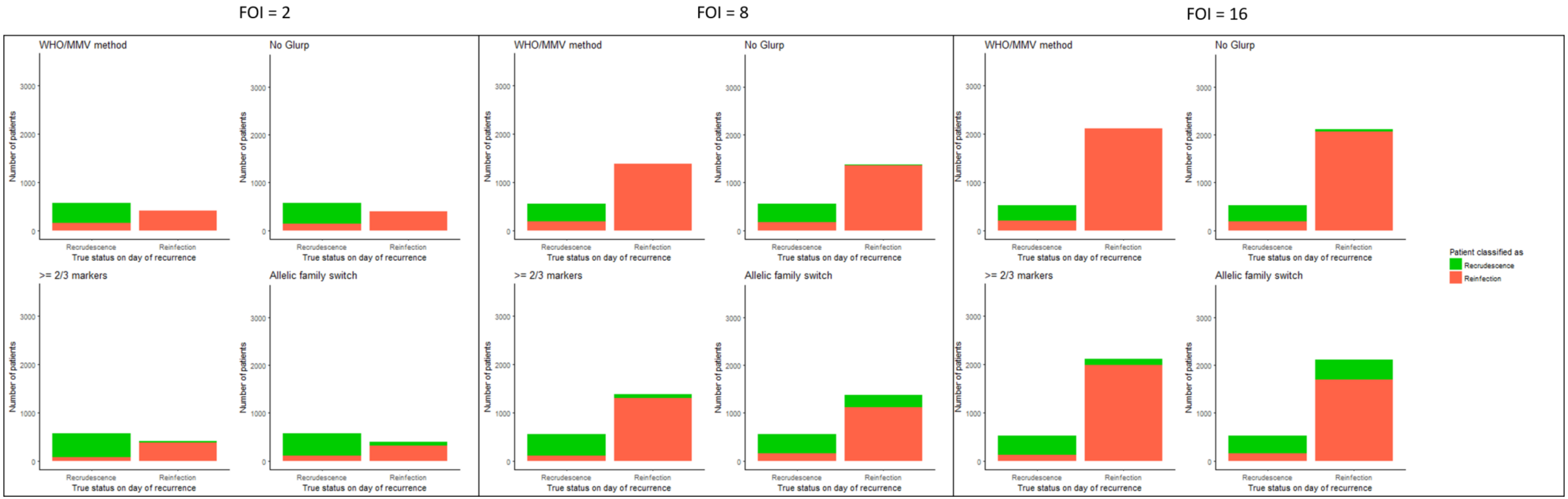

Figure S2 : Figure showing the ability of the various molecular correction algorithms to correctly classify patients with recurrent malaria. The data are for DHA-PPQ with a 42-day follow-up obtained with FOIs of 2, 8 and 16 (8 is identical to Figure 3 (main text)), showing how misclassification by each algorithm alters as FOI changes. The X-axis shows the true status of patients on the day of recurrence (i.e. reinfection or a recrudescence) and the colour-coding shows how these patients were classified by each algorithm.

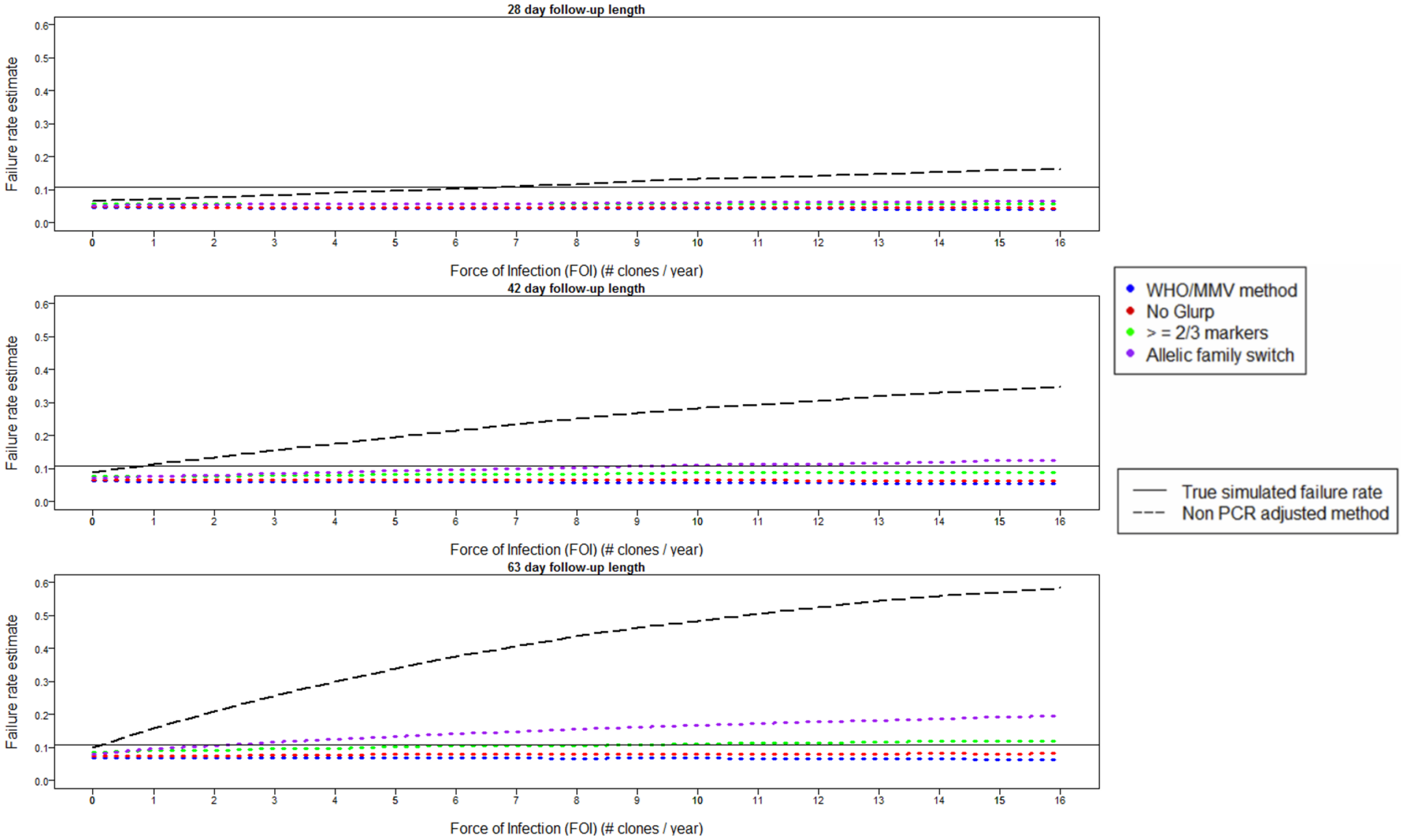
28 day follow-up length
42 day follow-up length
63 day follow-up length
Failure rate estimate
Force of Infection (FOI) (# clones / year)
WHO/MMV method
No Glurp
> = 2/3 markers
Allelic family switch
True simulated failure rate
Non PCR adjusted method

Figure S3: Analysis of simulated trial data for DHA-PPQ using a three compartment model (see Table S2) with follow-up lengths of (A) 28 days, (b) 42 days and (C) 63 days.

Estimated failure rates are shown for the different algorithms of molecular correction as a function of FOI and calculated using survival analysis.

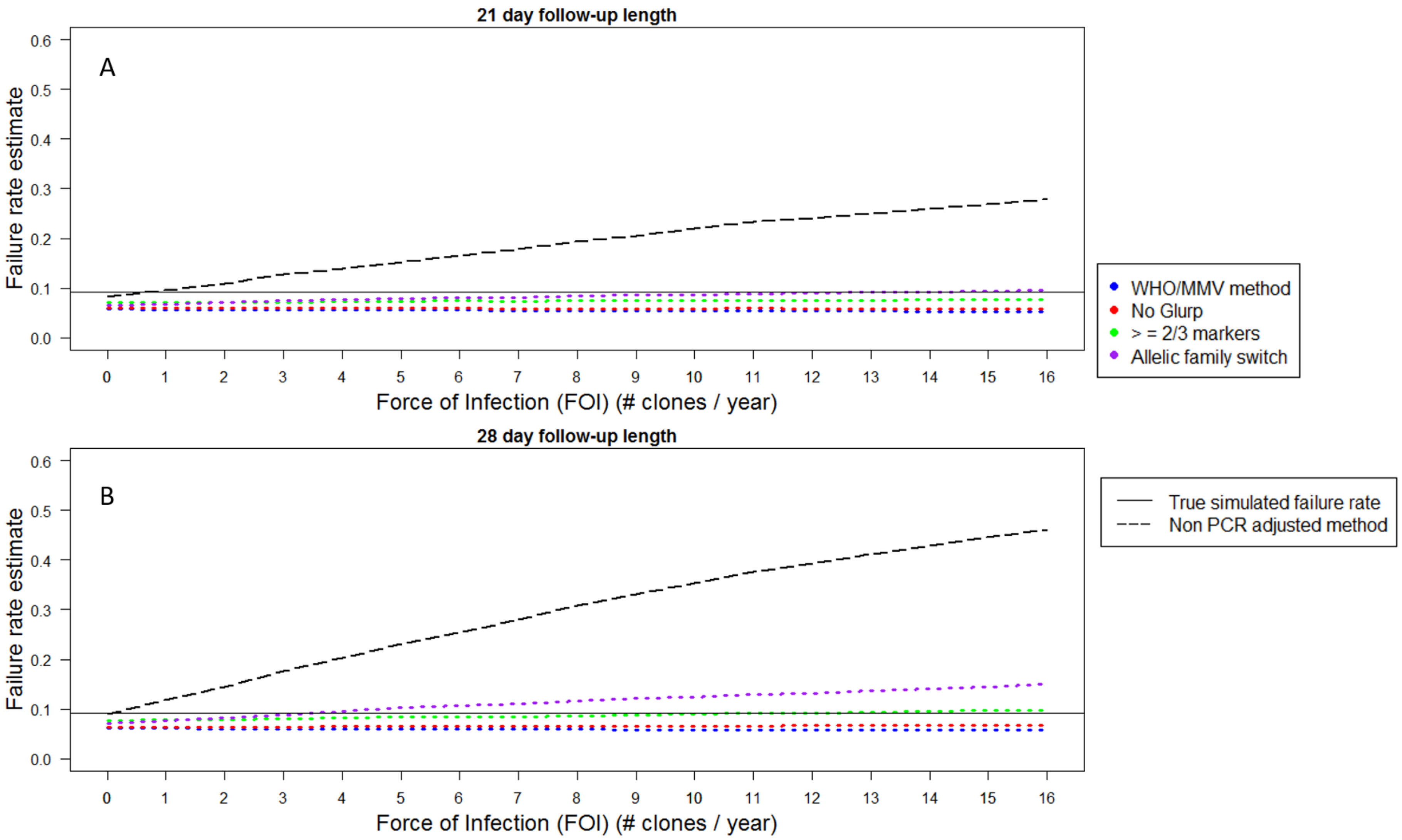

Figure S4 : Analysis of simulated trial data for failing AR-LF with follow-up lengths of 21 days (A) and 28 days (B). Estimated failure rates are shown for the different algorithms of molecular correction as a function of FOI and calculated using survival analysis.

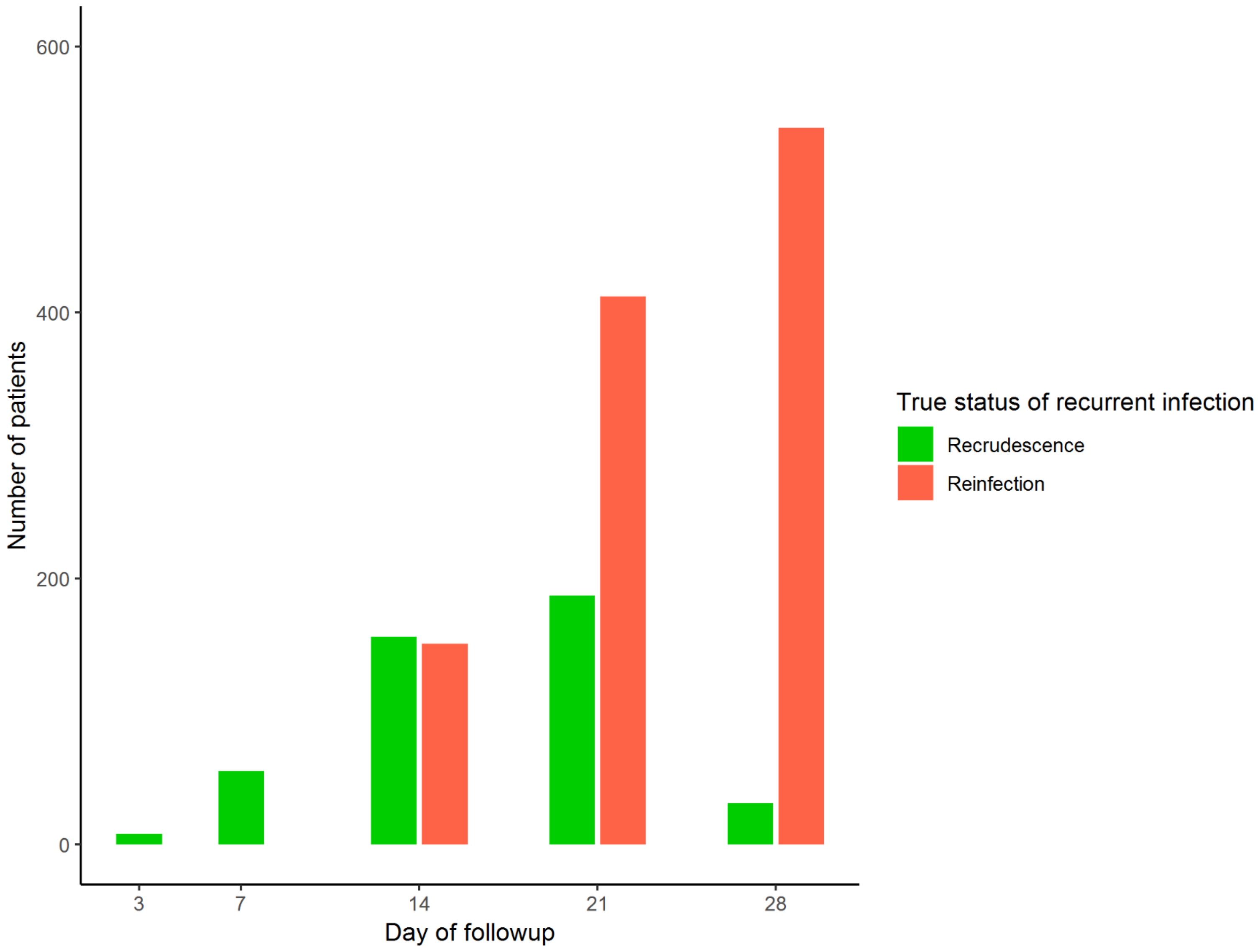

600
400
200
0
Number of patients
3
7
14
21
28
Day of followup
True status of recurrent infection
Recrudescence
Reinfection

Figure S5 : The true status of recurrent infections on each day of follow-up for a simulated trial of AR-LF with a true simulated failure rate of 9% and an FOI of 8. The total height of the bars indicates the number of recurrent infections detected on that day of follow-up, and the color-coding shows the number of those recurrent infectoins that were truly recrudescent or reinfections.

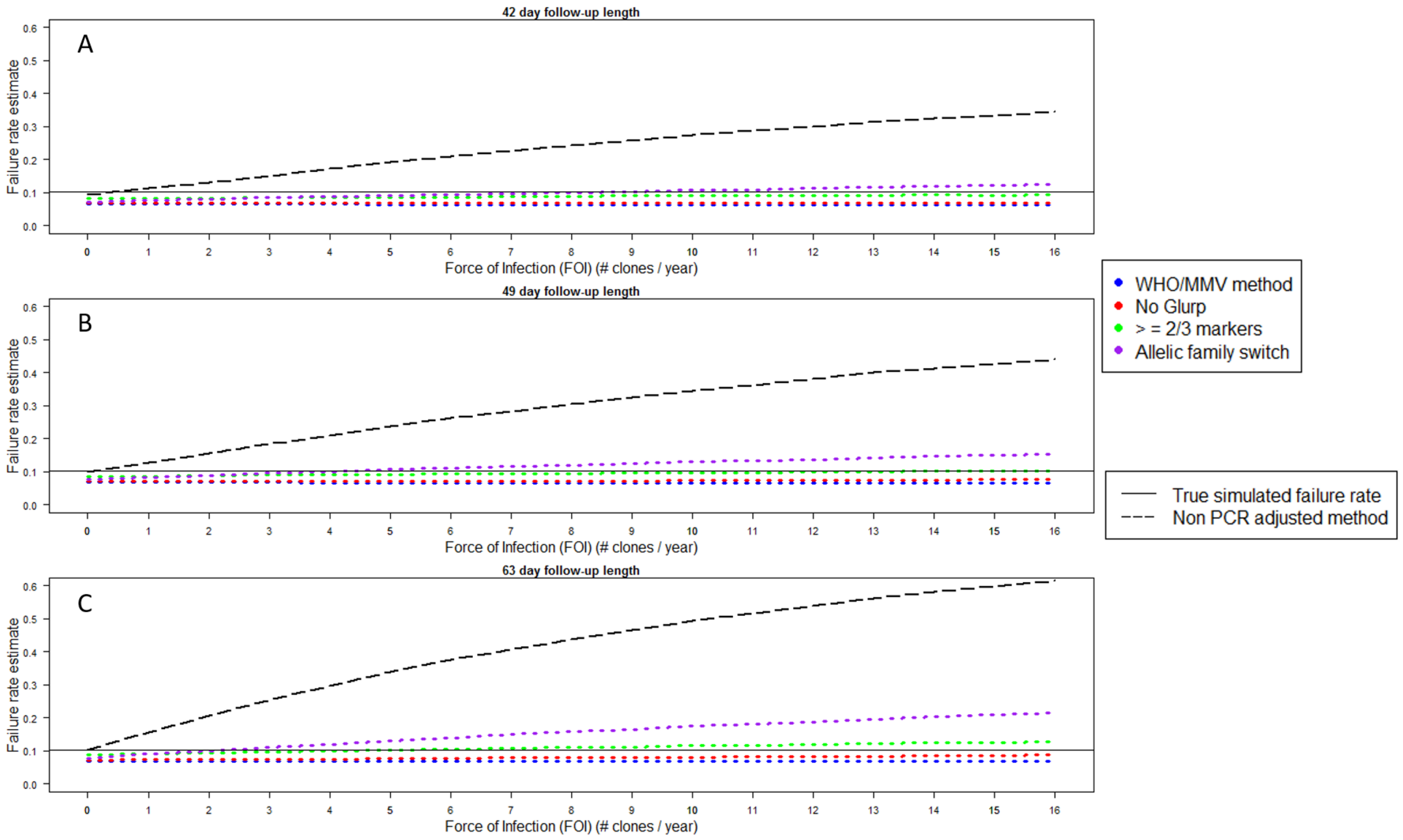

Figure S6 : Analysis of simulated trial data for failing AS-MQ with follow-up lengths of 42 days (A), 49 days (B) and 63 days (C). Estimated failure rates are shown for the different algorithms of molecular correction as a function of FOI and calculated using survival analysis.

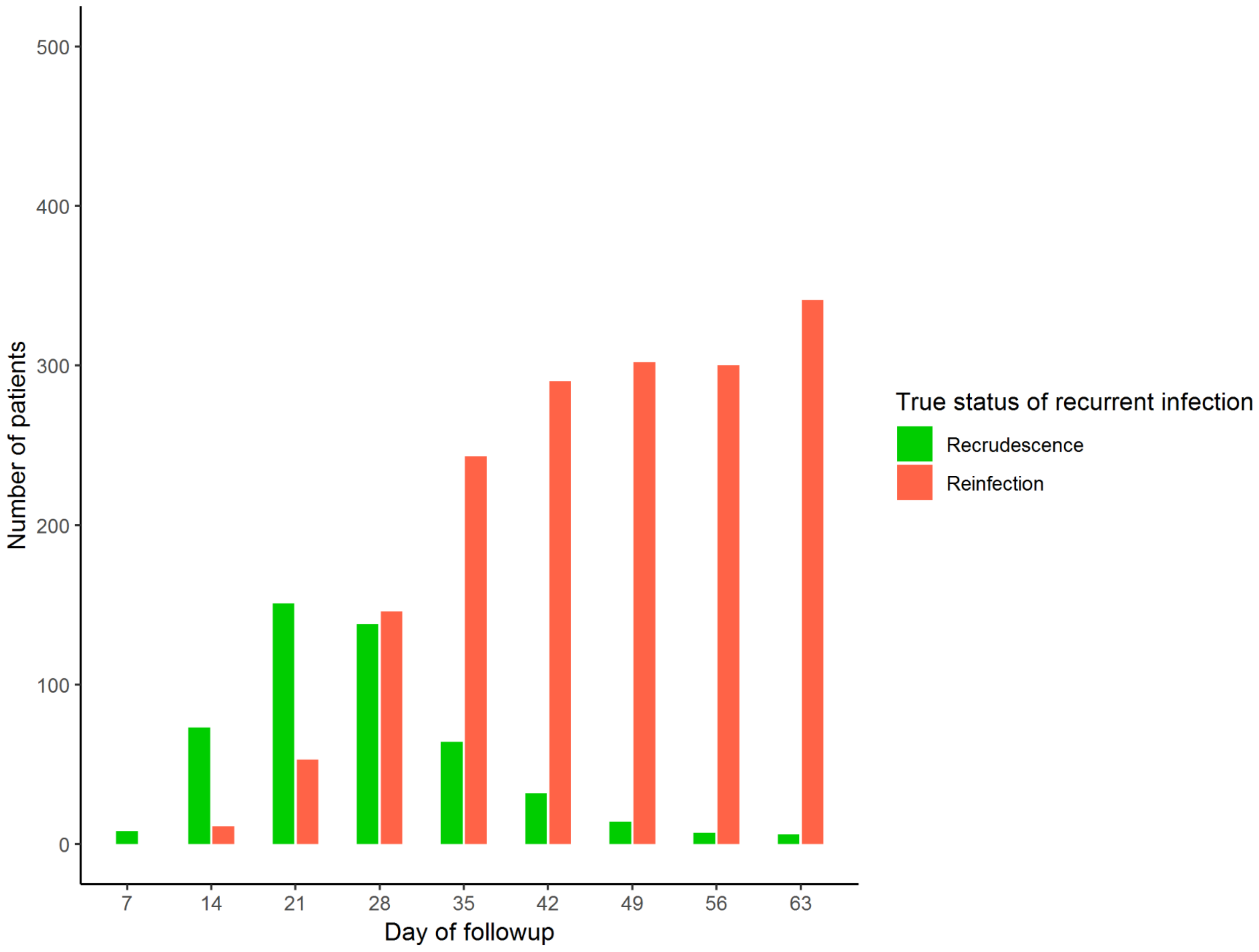

Number of patients
500
400
300
200
100
0
7
14
21
28
35
42
49
56
63
Day of followup
True status of recurrent infection
Recrudescence
Reinfection

Figure S7 : The true status of recurrent infections on each day of follow-up for a simulated trial of AS-MQ with a true simulated failure rate of 10% and an FOI of 8. The total height of the bars indicates the number of recurrent infections detected on that day of follow-up, and the color-coding shows the number of those recurrent infectoins that were truly recrudescent or reinfections.

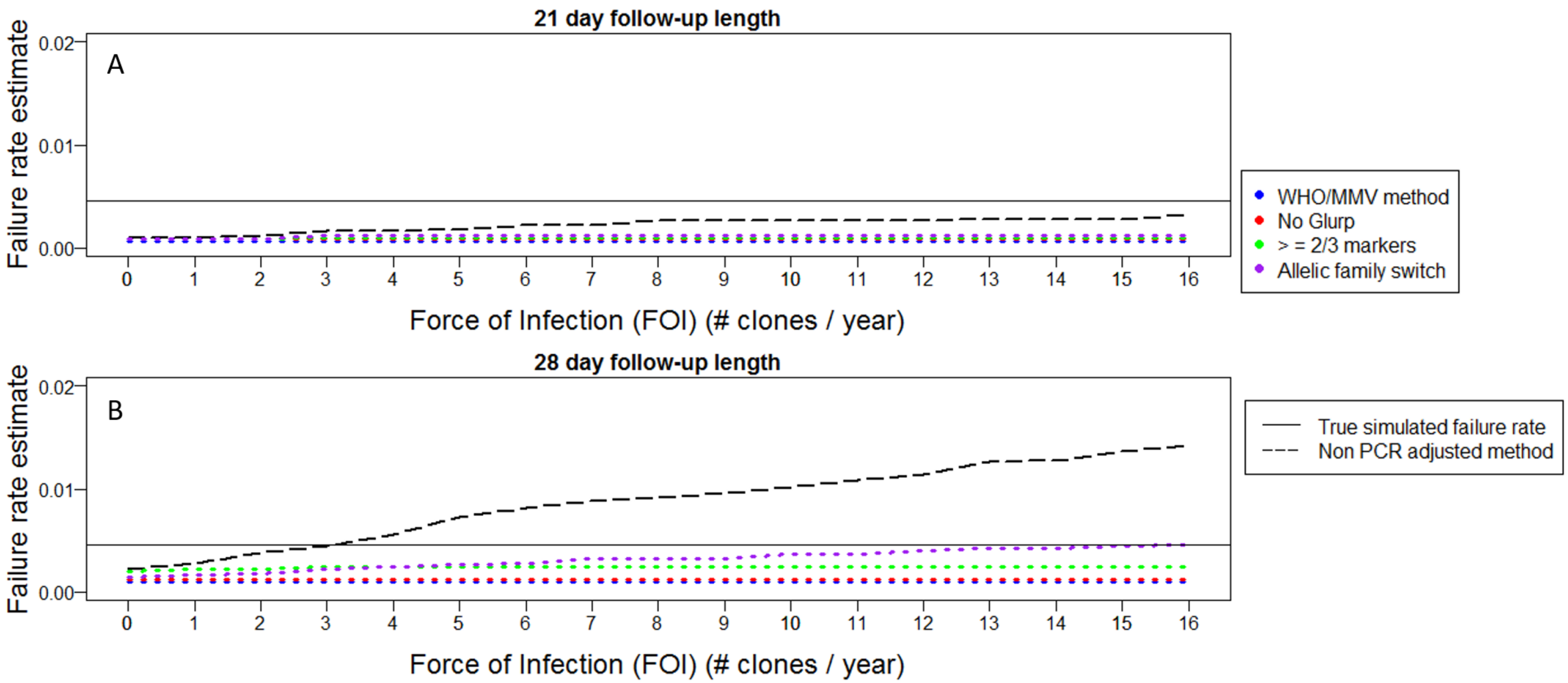

Figure S8 : Analysis of simulated trial data for effective AR-LF with follow-up lengths of 21 days (A) and 28 days (B). Estimated failure rates are shown for the different algorithms of molecular correction as a function of FOI and calculated using survival analysis.

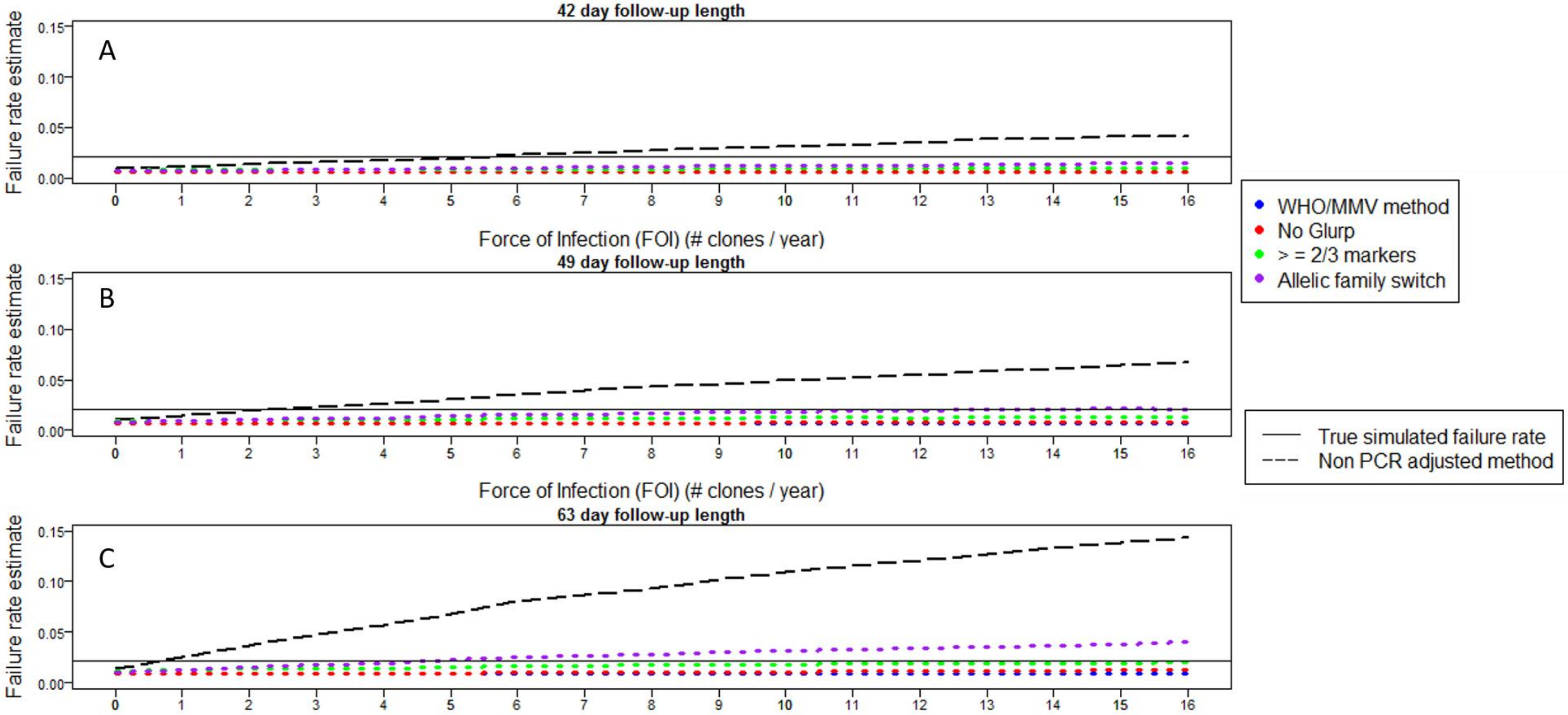

Figure S9 : Analysis of simulated trial data for effective AS-MQ with follow-up lengths of 42 days (A), 49 days (B) and 63 days (C). Estimated failure rates are shown for the different algorithms of molecular correction as a function of FOI and calculated using survival analysis.

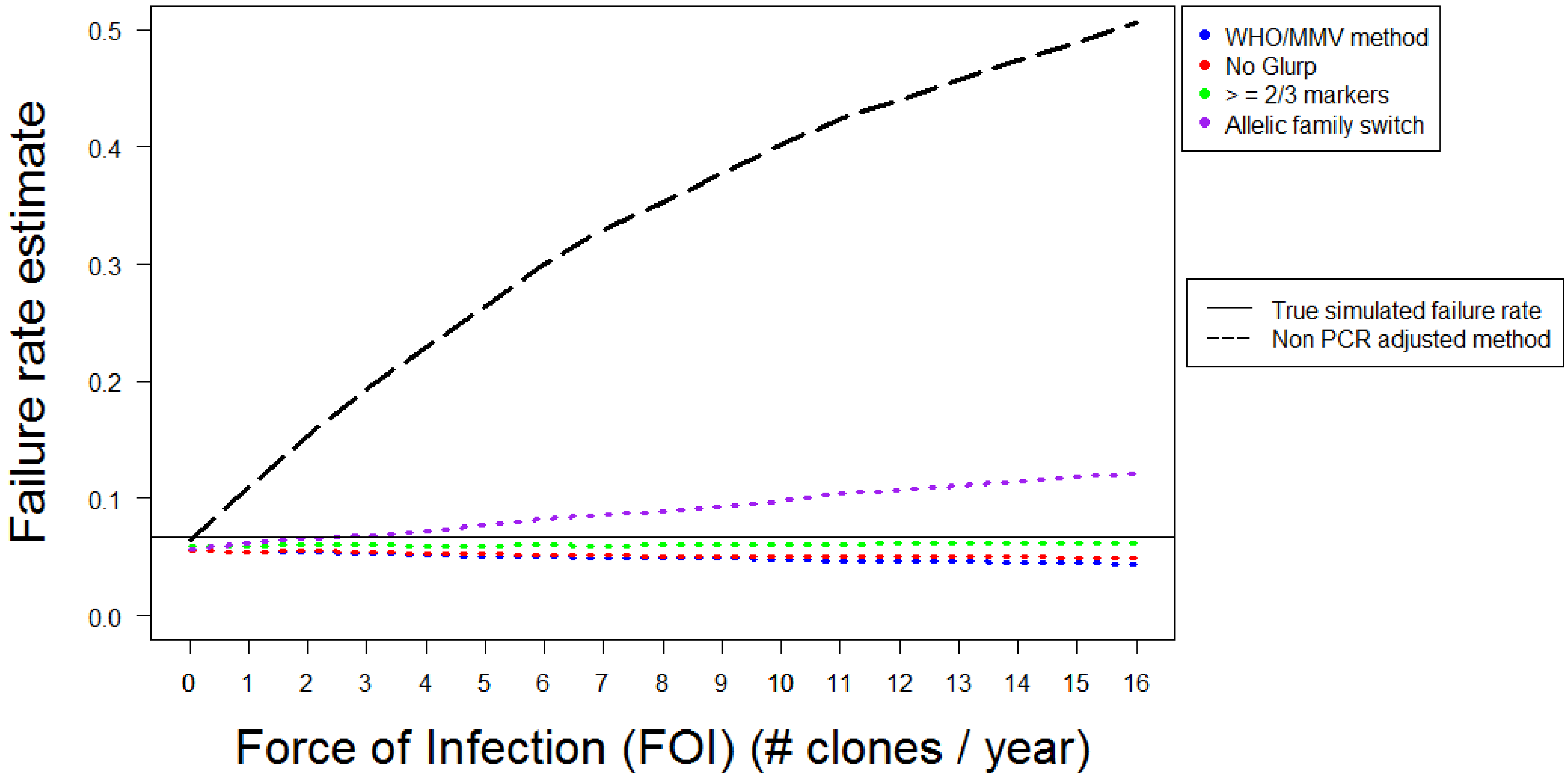

Figure S10: Analysis of simulated trial data for DHA-PPQ with a follow-up period of 42 days in a low MOI setting. Estimated failure rates are shown for the different algorithms of molecular correction as a function of FOI and calculated using survival analysis.

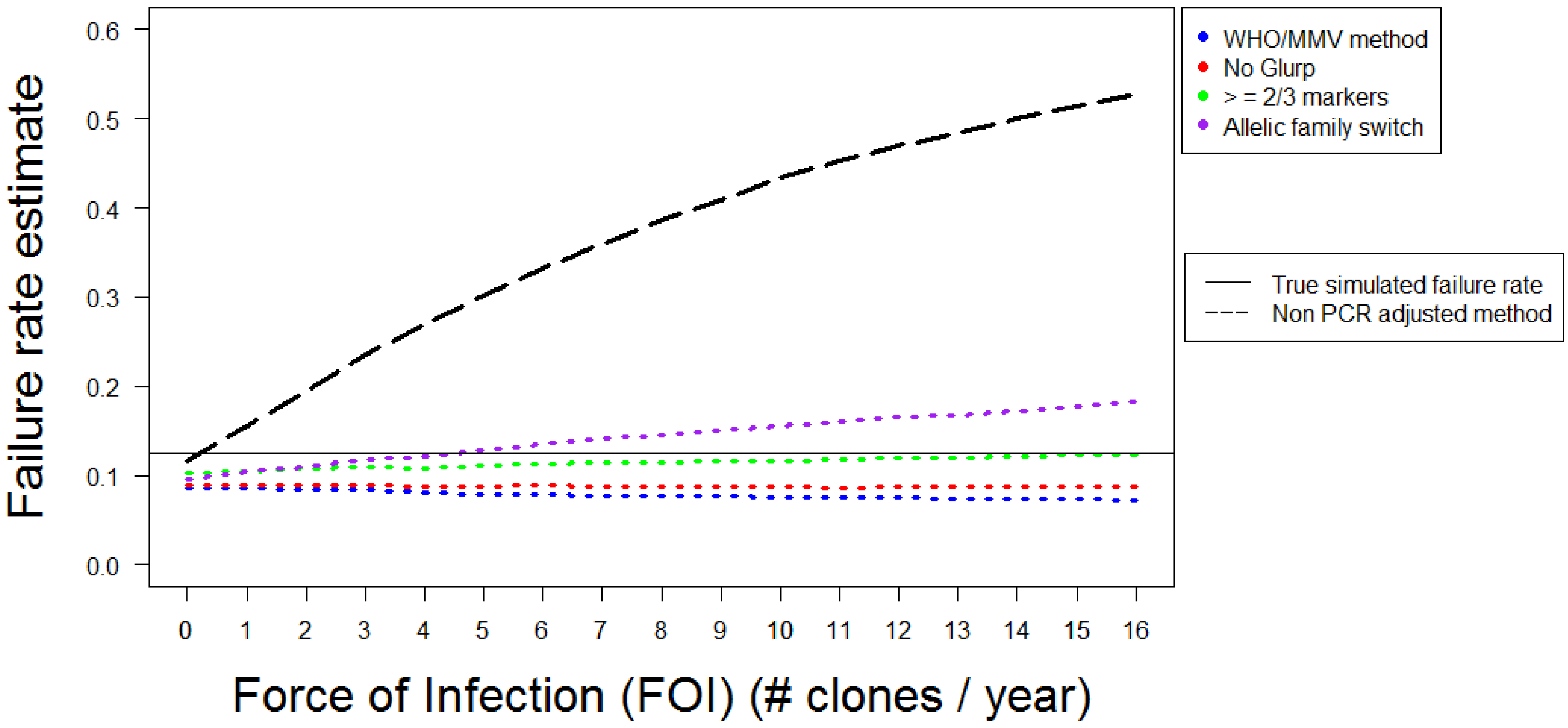

Figure S11: Analysis of simulated trial data for DHA-PPQ with a follow-up period of 42 days with the relative detectability of the longest allele to the shortest allele set to be 0.1:1. Estimated failure rates are shown for the different algorithms of molecular correction as a function of FOI and calculated using survival analysis.

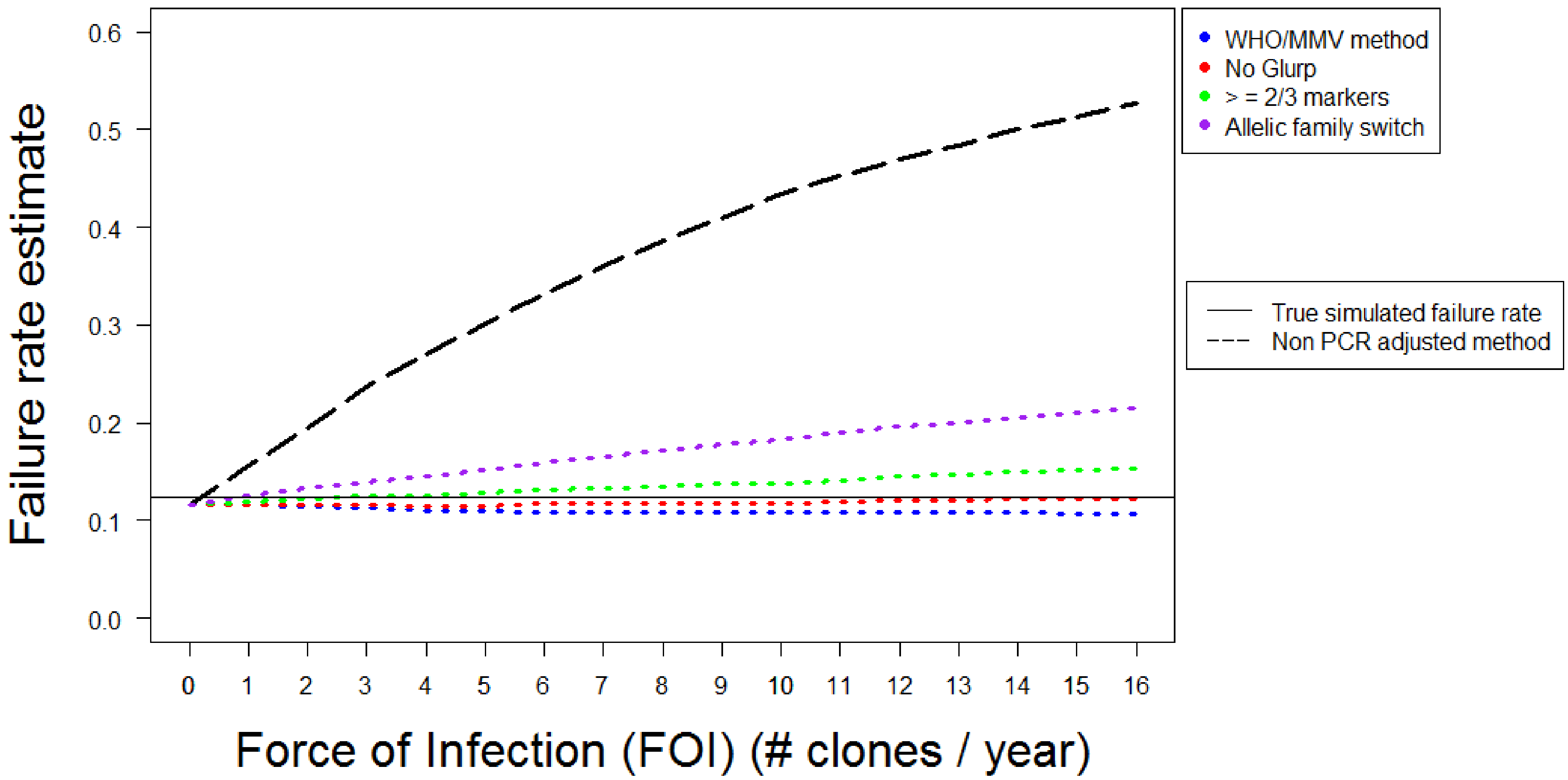

Figure S12: Analysis of simulated trial data for DHA-PPQ with a follow-up period of 42 days and a minority allele detection threshold of 0.05. Estimated failure rates are shown for the different algorithms of molecular correction as a function of FOI and calculated using survival analysis.

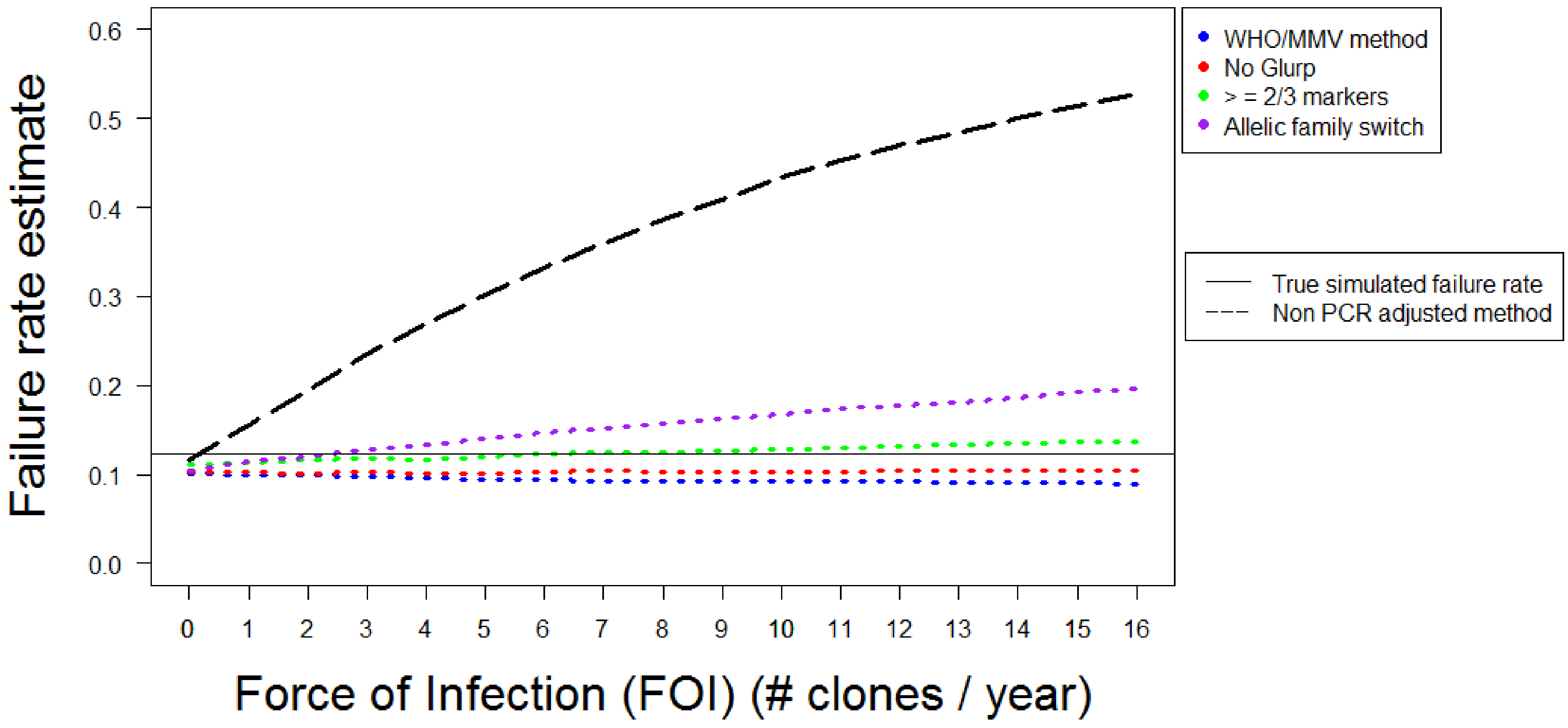

Figure S13: Analysis of simulated trial data for DHA-PPQ with a follow-up period of 42 days and a minority allele detection threshold of 0.2. Estimated failure rates are shown for the different algorithms of molecular correction as a function of FOI and calculated using survival analysis.

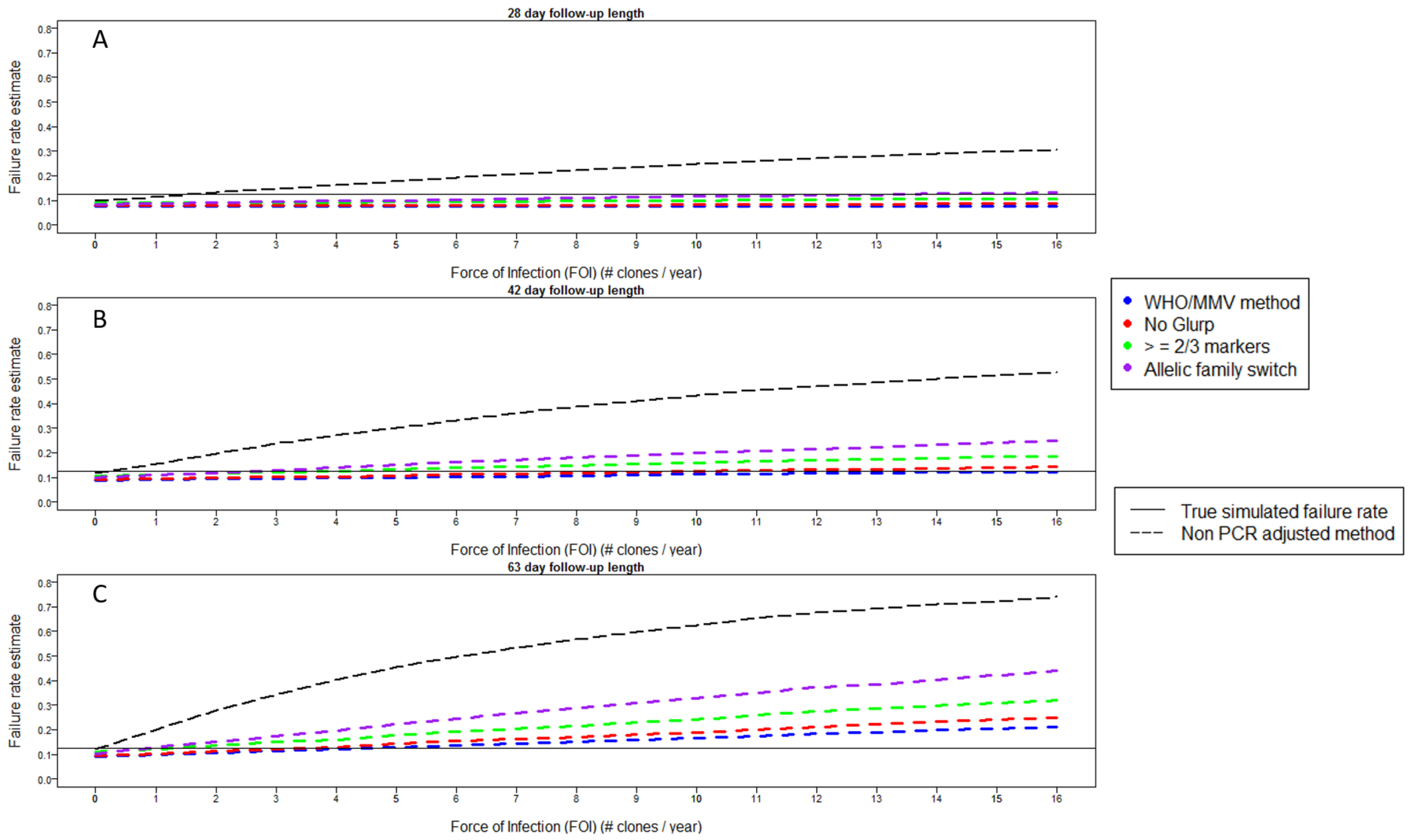

Figure S14: Analysis of simulated trial data for DHA-PPQ with follow-up lengths of 28 days (A), 42 days (B) and 63 days (C). Estimated failure rates are shown for the different algorithms of molecular correction as a function of FOI and calculated using the per protocol method.

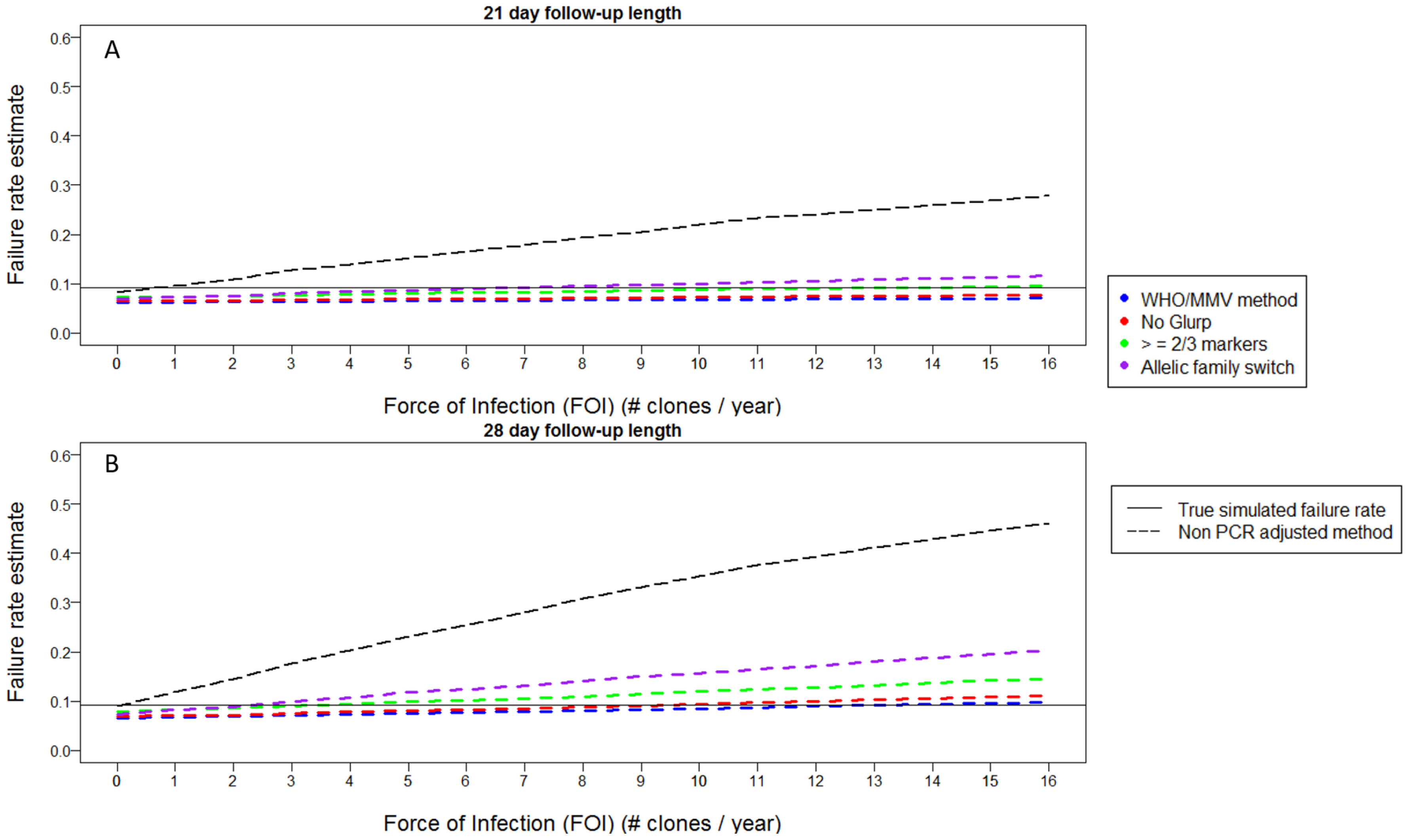

Figure S15: Analysis of simulated trial data for failing AR-LF with follow-up lengths of 21 days (A) and 28 days (B). Estimated failure rates are shown for the different algorithms of molecular correction as a function of FOI and calculated using the per protocol method.

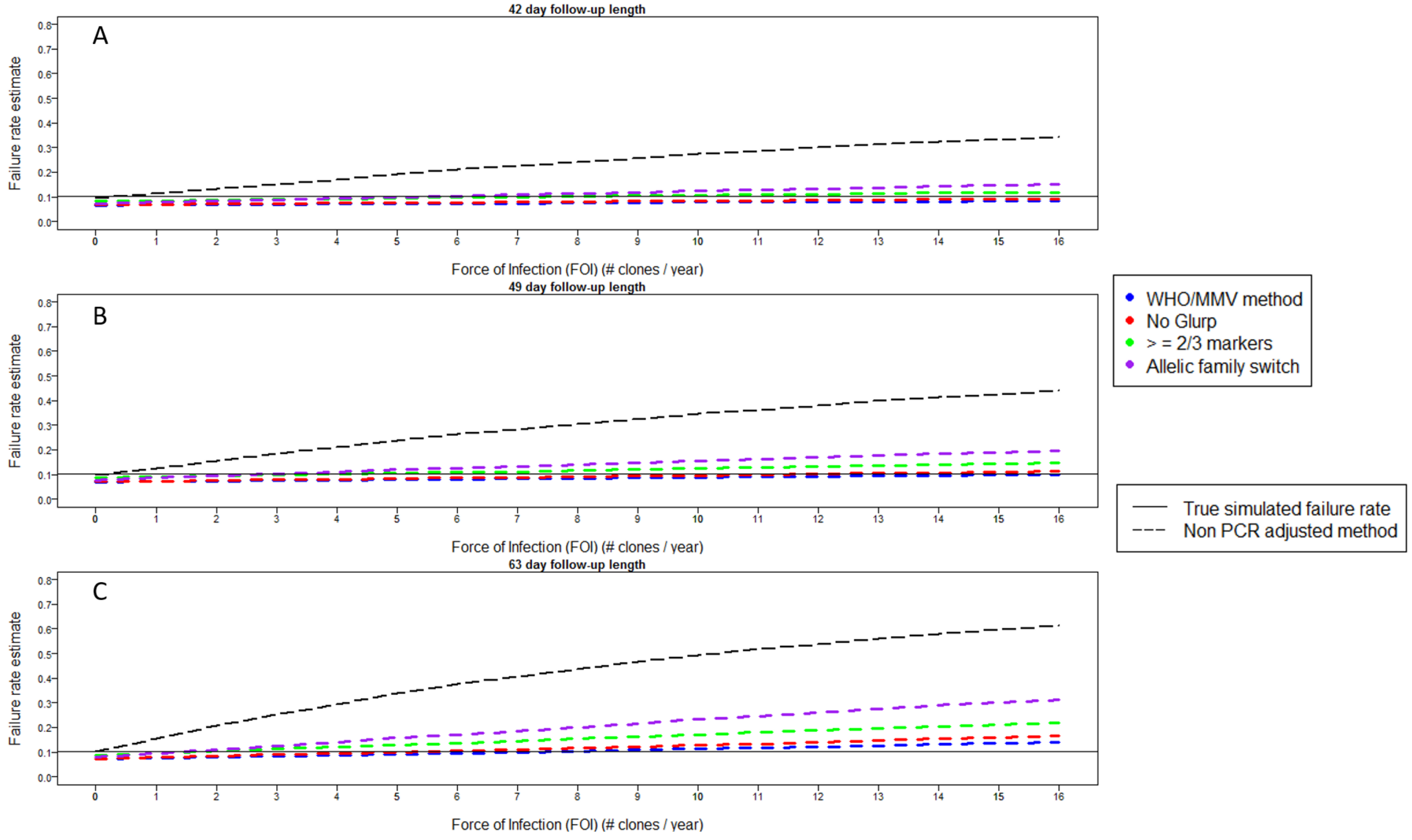

Figure S16 : Analysis of simulated trial data for failing AS-MQ with follow-up lengths of 42 days (A), 49 days (B) and 63 days (C). Estimated failure rates are shown for the different algorithms of molecular correction as a function of FOI and calculated using the per protocol method.

References

1. Kay K, Hastings IM. 2013. Improving Pharmacokinetic-Pharmacodynamic Modeling to Investigate Anti-Infective Chemotherapy with Application to the Current Generation of Antimalarial Drugs. PLoS Comput Biol 9:e1003151.
2. Kay K, Hastings IM. 2015. Measuring windows of selection for anti-malarial drug treatments. Malaria Journal 14:292.
3. Winter K, Hastings IM. 2011. Development, evaluation, and application of an in silico model for antimalarial drug treatment and failure. Antimicrob Agents Chemother 55:3380-92.
4. Kay K, Hodel EM, Hastings IM. 2014. Improving the role and contribution of pharmacokinetic analyses in antimalarial drug clinical trials. Antimicrob Agents Chemother 58:5643-9.
5. Kay K, Hodel EM, Hastings IM. 2015. Altering Antimalarial Drug Regimens May Dramatically Enhance and Restore Drug Effectiveness. Antimicrobial Agents and Chemotherapy 59:6419-6427.
6. Saunders DL, Vanachayangkul P, Lon C. 2014. Dihydroartemisinin–Piperaquine Failure in Cambodia. New England Journal of Medicine 371:484-485.
7. World Health Organization. 2009. Methods for surveillance of antimalarial drug efficacy.
8. Tarning J, Rijken MJ, McGready R, Phyo AP, Hanpithakpong W, Day NP, White NJ, Nosten F, Lindegardh N. 2012. Population pharmacokinetics of dihydroartemisinin and piperaquine in pregnant and nonpregnant women with uncomplicated malaria. Antimicrob Agents Chemother 56:1997-2007.
9. Stepniewska K, Taylor W, Sirima S, Ouedraogo E, Ouedraogo A, Gansane A, Simpson J, Morgan C, White N, Kiechel J-R. 2009. Population pharmacokinetics of artesunate and amodiaquine in African children. Malaria Journal 8:200.

10. Hong KB. 2014. Pharmacological modelling of the efficacy and safety of fixed-dose versus non-fixed dose combinations of the antimlarial drug artesunate-amodiaquine. MSc. Liverpool School of Tropical Medicine, Liverpool.
11. Htay MNN. 2014. Pharamcological modelling of intermittent preventative treatment in pregnancy with sulpadoxine-pyrimethamine. MSc. Liverpool School of Tropical Medicine, Liverpool.
12. Mueller I, Schoepflin S, Smith TA, Benton KL, Bretscher MT, Lin E, Kiniboro B, Zimmerman PA, Speed TP, Siba P, Felger I. 2012. Force of infection is key to understanding the epidemiology of Plasmodium falciparum malaria in Papua New Guinean children. Proc Natl Acad Sci U S A 109:10030-5.
13. Felger I, Maire M, Bretscher MT, Falk N, Tiaden A, Sama W, Beck H-P, Owusu-Agyei S, Smith TA. 2012. The Dynamics of Natural Plasmodium falciparum Infections. PLOS ONE 7:e45542.
14. Smith T, Maire N, Dietz K, Killeen G, Vounatsou P, Molineaux L. 2006. Relationships between the entomological inoculation rate and the force of infection for Plasmodium falciparum malaria. Am J Trop Med Hyg 75:11 - 18.
15. Dahal P, Simpson JA, Dorsey G, Guérin PJ, Price RN, Stepniewska K. 2017. Statistical methods to derive efficacy estimates of anti-malarials for uncomplicated Plasmodium falciparum malaria: pitfalls and challenges. Malaria Journal 16:430.
16. Lerch A, al. e. 2018. Development of amplicon deep sequencing markers and data analysis pipeline for genotyping multi-clonal malaria infections. BMC Genomics in press.
17. Plucinski MM, Morton L, Bushman M, Dimbu PR, Udhayakumar V. 2015. Robust Algorithm for Systematic Classification of Malaria Late Treatment Failures as Recrudescence or Reinfection Using Microsatellite Genotyping. Antimicrob Agents Chemother 59:6096-100.
18. Greenhouse B, Myrick A, Dokomajilar C, Woo JM, Carlson EJ, Rosenthal PJ, Dorsey G. 2006. VALIDATION OF MICROSATELLITE MARKERS FOR USE IN GENOTYPING POLYCLONAL PLASMODIUM FALCIPARUM INFECTIONS. The American journal of tropical medicine and hygiene 75:836-842.

19. Plucinski MM, Morton L, Bushman M, Dimbu PR, Udhayakumar V. 2015. Robust algorithm for systematic classification of malaria late treatment failures as recrudescence or reinfection using microsatellite genotyping. Antimicrob Agents Chemother 59.
20. Hodel EM, Kay K, Hayes DJ, Terlouw DJ, Hastings IM. 2014. Optimizing the programmatic deployment of the anti-malarials artemether-lumefantrine and dihydroartemisinin-piperaquine using pharmacological modelling. Malar J 13:138.
21. Kay K, Hodel EM, Hastings IM. 2015. Altering antimalarial drug regimens may dramatically enhance and restore drug effectiveness. Antimicrobial Agents and Chemotherapy doi:10.1128/aac.00482-15.
22. Staehli Hodel EM, Guidi M, Zanolari B, Mercier T, Duong S, Kabanywanyi AM, Ariey F, Buclin T, Beck H-P, Decosterd LA, Olliaro P, Genton B, Csajka C. 2013. Population pharmacokinetics of mefloquine, piperaquine and artemether-lumefantrine in Cambodian and Tanzanian malaria patients. Malaria journal 12:235-235.
